# Acousto-holographic reconstruction of whole-cell stiffness maps

**DOI:** 10.1038/s41467-022-35075-x

**Published:** 2022-11-29

**Authors:** Rahmetullah Varol, Zeynep Karavelioglu, Sevde Omeroglu, Gizem Aydemir, Aslihan Karadag, Hanife E. Meco, Ali A. Demircali, Abdurrahim Yilmaz, Gizem C. Kocal, Gulsum Gencoglan, Muhammed E. Oruc, Gokhan B. Esmer, Yasemin Basbinar, Sahin K. Ozdemir, Huseyin Uvet

**Affiliations:** 1 Department of Mechatronics Engineering, Yildiz Technical University, Istanbul, 34349 Turkey; 2 Bioengineering Department, Yildiz Technical University, Istanbul, 34220 Turkey; 3 Paul Scherrer Institute, Villigen, 5232 Switzerland; 4 Chemical Engineering Department, Gebze Technical University, Kocaeli, 41400 Turkey; 5 Oncology Institute, Dokuz Eylul University, Izmir, 35330 Turkey; 6 Department of Dermatology, Istinye University, Istanbul, 34722 Turkey; 7 Electrical and Electronics Engineering Department, Marmara University, Istanbul, 34722 Turkey; 8 Department of Engineering Science and Mechanics, The Pennsylvania State University, University Park, PA 16802 USA; 9 Present Address: Universität der Bundeswehr München, Munich, 85577 Germany; 10 Present Address: Eidgenössische Technische Hochschule Zürich, Zürich, 8092 Switzerland; 11 Present Address: Hamlyn Centre, Imperial College London, London, SW7 2AZ UK

**Keywords:** 3-D reconstruction, Imaging and sensing, Imaging techniques, Atomic force microscopy

## Abstract

Accurate assessment of cell stiffness distribution is essential due to the critical role of cell mechanobiology in regulation of vital cellular processes like proliferation, adhesion, migration, and motility. Stiffness provides critical information in understanding onset and progress of various diseases, including metastasis and differentiation of cancer. Atomic force microscopy and optical trapping set the gold standard in stiffness measurements. However, their widespread use has been hampered with long processing times, unreliable contact point determination, physical damage to cells, and unsuitability for multiple cell analysis. Here, we demonstrate a simple, fast, label-free, and high-resolution technique using acoustic stimulation and holographic imaging to reconstruct stiffness maps of single cells. We used this acousto-holographic method to determine stiffness maps of HCT116 and CTC-mimicking HCT116 cells and differentiate between them. Our system would enable widespread use of whole-cell stiffness measurements in clinical and research settings for cancer studies, disease modeling, drug testing, and diagnostics.

## Abstract (web summary)

Traditional methods for cell stiffness measurements are limited by long processing times and unsuitability for multiple cell analysis. Here, the authors demonstrate a fast technique based on acoustic stimulation and holographic imaging to reconstruct whole-cell stiffness maps of individual and multiple cells.

## Introduction

The mechanical properties, stability, and integrity of biological cells are provided by the cytoskeleton, which develops during cell differentiation and plays significant roles in many cellular functions. Transformational changes in cytoskeleton affect a cell’s mechanical behavior, which is closely related to its cellular architecture and regulation of biological functions such as cell proliferation^1^, migration^2^, and motility^3^. Studies have associated such alterations on the mechanical properties of cells with the pathogenesis and progression of the various diseases^4–6^. Transformation of cancer cells from normal to malignant form is also characterized by changes in their mechanical properties^7^. Cancer cells tend to have a lower mechanical stiffness compared to normal cells^8^; metastatic cells exhibit a softer profile than benign cells^9^; and cancer cells send signals in the form of secreted extracellular vesicles that can affect the mechanical conditions of other cells^10^. Therefore, studying and assessing mechanical properties of cancer cells at the single-cell level may shed light on cancer progression and its mechanisms.

Cell stiffness is an important and widely studied probe of mechanical properties linked to many biological functions, intracellular tensional forces, cytoskeletal prestress, and cytoskeleton structure. It is greatly affected by the intracellular tensional forces, cytoskeletal prestress, and cytoskeleton structure. Accurate measurement of a cell’s stiffness provides insights into various physiological and pathological processes and enables the assessment of cell viability, proliferation, differentiation, migration, and invasion, all of which are involved in disease development. Over the years, many different techniques have been developed for cell stiffness measurements, such as atomic force microscopy (AFM)^4^, optical trapping (OT)^11^, magnetic twisting cytometry^12^, deformation cytometry^13^, particle-tracking microrheology^14^, micropipette aspiration^15^, parallel-plate rheometry^16^, cell monolayer rheometry^16^, and optical stretching^17^, just to name a few. There are still more grounds to cover as these techniques usually yield varying results up to ten-fold. Furthermore, many of these methods typically rely on heavy preconditioning of the samples, such as fixation or attachment of various surface markers. These procedures compromise the viability of cells and do not allow for repeatable measurements over a single sample^16^.

In parallel to these techniques, quantitative phase imaging methods^18,19^ such as digital holographic microscopy (DHM)^20–22^, holographic tomography^23^, and full-field interferometry^24^ have also been developed and used for cellular imaging: DHM for live cell imaging^25^, holographic tomography for resolving inner structure of single cells^26^, and full field interferometry for capturing nanoscale morphological changes^24^ have been already demonstrated. DHM, in particular, presents unique advantages compared to other imaging modalities such as the ability for autofocusing^27^, virtual staining^28^, or descattering^29^. An interesting and uniquely different approach than the above was introduced in Kang et al. where cell stiffness is measured by monitoring the changes in the resonance frequency of a cantilever-based microfluidic channel due to the scattering of the cantilever’s acoustic field from a cell’s surface^30^. Although this method has allowed single-cell stiffness measurements with high temporal resolution over long periods, it requires accurate determination of cell’s mass distribution along the channel using bright-field images or a priori information of cell’s shape, and it only provides an average value for stiffness but does not provide stiffness map which is important to understand the stiffness of subcellular components. Another promising study is the one by Hwang et al. who used acoustic trapping to determine cell deformation and hence cell’s stiffness^31^. However, this method, too, provides an average cell stiffness rather than a stiffness map.

Among many methods for cell stiffness measurements, AFM and OT methods set the gold standard and have been widely utilized as research tools. AFM-based systems have been used to determine stiffness maps using different deformation models such as the Hertzian model which gives good results only for small deformations^32^, mechanical characteristics of individual live and dead cells^33^, stiffness levels of breast cancer cells^5^, and morphological and mechanical changes induced by external chemical and physical perturbations^34^. AFM can be used for both subcellular and whole-cell measurements by properly choosing the number of points on which the cell membrane is indented. OT-based methods, on the other hand, allow for measuring the whole-cell stiffness of suspended individual cells by determining their elastic modulus through a dual-beam optical trap that induces mechanical stress on the cells^35^. Despite their superior resolution and performance, AFM and OT techniques do not lend themselves for widespread clinical use due to their limitations attributed to difficult handling, time-consuming and slow operation; costly, bulky, and complex setups; impossibility of measurements on large number of cells; and the possibility of cell damage (i.e., cells may suffer from mechanical damage in AFM and from heating or thermal damage due to prolonged exposure in OT). In addition, AFM measurements are significantly affected by the geometry and size of the indenter tip as well as the location of the contact point. Here, we introduce a simple, label-free, fast, and non-invasive method based on acousto-holographic imaging that allows for high-resolution measurement of stiffness distribution over the membrane of single and multiple cells in the same environment. In this method, cell membrane is stimulated using periodic acoustic pressure waves and the membrane deformation is measured via a digital holographic microscope.

**Fig. 1:**
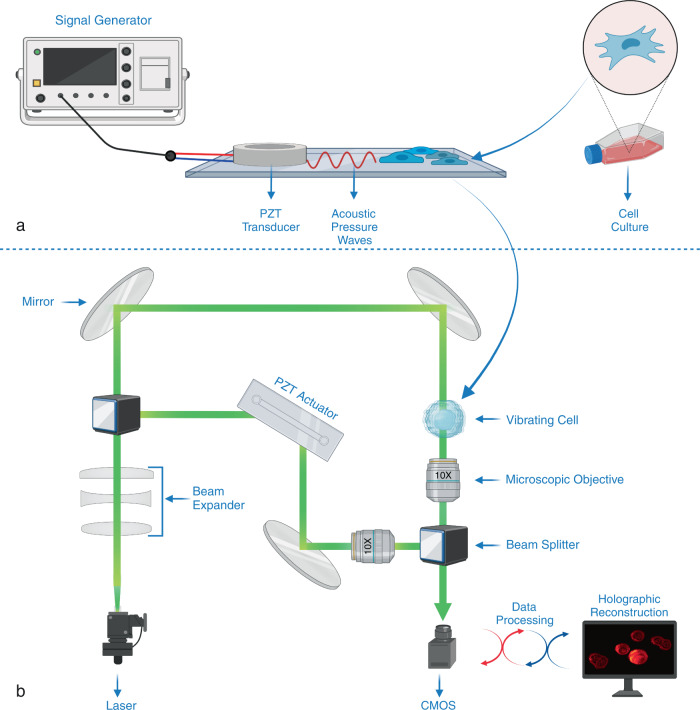
Acousto-holographic cell stiffness measurement. **a** Compression and deformation induced on a cell with a low intensity bulk acoustic wave is measured by an interferometric imaging system to obtain whole-cell stiffness distribution. A PDMS fluidic chamber consisting of a PZT transducer, placed between a PDMS layer and a glass substrate it is bonded, is used to stimulate the cells in the chamber with high-frequency acoustic signals. **b** Holographic imaging system is composed of a Mach-Zehnder interferometer whose output port is monitored with a CMOS detector (see “Methods”). Light from a 527 nm He-Ne laser is divided into two by a beamsplitter (BS) and sent to two arms of the interferometer. The cell medium is placed in one of the arms, and the phase difference between the arms was varied using a high-frequency piezo actuator. Optical fields from both arms of the interferometer are recombined at a second BS, and one of the BS outputs is monitored with a high-speed CMOS camera that records the interference pattern. The acquired interferograms are processes to reconstruct stiffness maps (see “Methods” and  Supplement for details). Created with BioRender.com.

Here, we present a simple and versatile system that combines acoustics and optics for the measurement and mapping of cell stiffness. In this system, a cell medium is stimulated by surface acoustic waves generated by a lead zirconate titanate (PZT) transducer, and the mechanical response of the cells in the medium are measured using DHM that employs a phase shifting inline Mach–Zehnder interferometer and a high-speed complementary metal-oxide-semiconductor (CMOS) camera (Fig. 1a). Small patches of the captured interferogram are sampled at a rate of 1 kHz to reconstruct the morphological transformations of the cell (see “Methods” for the details of the system and reconstruction algorithm). Cell stiffness was calculated using a Hertzian elasticity model^36^ through the association of acoustic pressure with cellular membrane deflection. In this model, the sample is approximated as an isotropic and linear elastic solid occupying an infinitely extending half space. The indentation due to the acoustic pressure is measured through the variation of the cellular thickness at the measured point. Our method allows for analyzing individual and multiple cells in a medium without directly contacting the cells. Thus, cell viability is not damaged by the possible cell-probe interactions, and cells can be examined in their environment. This opens new possibilities and opportunities in the study of cell biomechanics for applications in a wide range of areas including but not limited to cancer research and drug efficacy evaluation.

## Results

### Validation of the Method

We first tested the performance of our acousto-holographic imaging system in comparison with AFM measurements on reference polyacrylamide (PAA) microbeads with an average diameter $$ < 45$$ µm (Bio-Gel P-2 Gel #1504118), agarose microbeads with diameters in the range $$20-50$$ µm (Agarose Bead Technologies #A-1040F-250) and polystyrene (PS) microbeads with an average diameter $$5\pm 0.3$$ µm and an average stiffness $$1.05\pm 0.1$$ GPa (Thermo Scientific Duke Standards, 2005ATS). PAA and agarose beads are widely used in the literature as reference particles due to their characteristics that mimic cell elasticity, size, and shape^26,37^. PS particles in this study are used to check the accuracy of our acousto-holographic system with vendor provided size and stiffness values as well as to demonstrate its use for other types of reference particles. AFM and acousto-holographic measurements were performed in cell culture medium after the PAA and agarose microbeads were adhered to a glass surface using Cell-Tak (Corning 354240).

**Fig. 2:**
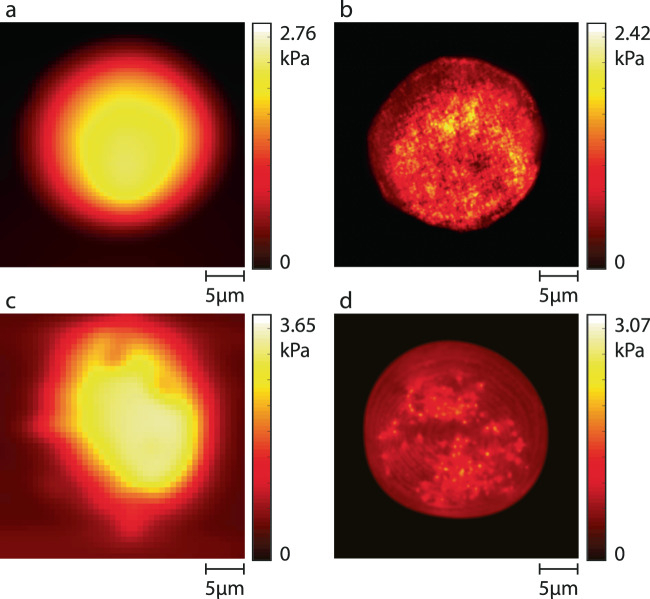
Stiffness measurement of microbeads using acousto-holographic imaging system and AFM. Stiffness maps of **a**, **b** PAA and **c**, **d** agarose microbeads obtained from measurement with **a**, **c** an AFM, and **b**, **d** the proposed acousto-holographic imaging system. Average stiffness values measured for the microbeads shown here are: **a** 1.79 kPa (PAA microbeads with AFM), **b** 1.52 kPa (PAA microbeads with acousto-holography), **c** 2.26 kPa (agarose microbeads with AFM), and **d** 2.06 kPa (agarose microbeads with acousto-holography). See the “Methods” and the Supplement for the details of reconstruction of stiffness maps and measurement results for PS microbeads.

AFM (Nanosurf Flex Axiom) was operated in the force modulation mode using a DNP-S10 cantilever (length: 120 µm; width: 25 µm; natural frequency: 65 kHz; and stiffness: 0.35 N/m) with a silicon tip of 10 nm in radius. A single scan was conducted on a 25 µm × 25 µm area with a resolution of 256 × 256 points with a static force of 20 nN (typical settings used in the literature for AFM-based cellular stiffness measurements^38^). Acousto-holographic measurements were performed at 1 kHz, and the acquired interferograms were processed to obtain the holographic images and thickness maps, which were then used to reconstruct displacement waveforms. Elastic modulus of the microbeads and their stiffness distribution were obtained from the displacement waveforms using a theoretical model (see Supplement and “Methods”). From acousto-holographic measurement, we found the stiffness and thickness distribution for an ensemble of 50 PS microbeads as $1.05\pm 0.11$ GPa and $4.94\pm 0.12$ µm, respectively (see the Supplement Fig. S1 for the holographic image, thickness map, displacement, and the stiffness distribution of the PS microbeads). These are in good agreement with the values of $1\pm 0.1$ GPa and $5\pm 0.05$ µm provided by the vendor and are used to calibrate and validate the stiffness measurements obtained from the acousto-holographic imaging system. Stiffness values for PAA and agarose particles were not available from the vendor; therefore, we validated the performance of our system by comparing the results with those from AFM measurements (Fig. 2). It is clearly seen that the acousto-holographic system provides a stiffness map which helps identify local stiffness over the particle surface. Our AFM measurements failed to provide such a resolved map of local stiffness distribution. Using the stiffness map obtained with AFM (Fig. 2a, c), we estimated the average stiffness as 1.79 kPa and 2.26 kPa for the measured PAA and agarose particle, respectively. The stiffness maps obtained from acousto-holographic measurements (Fig. 2b, d) yielded the average stiffness values as 1.59 kPa and 2.11 kPa, respectively, for PAA and agarose microbeads, which are in good agreement with the values measured with AFM. The discrepancy may be due to the particle-to-particle variation in the stiffness values.

**Fig. 3:**
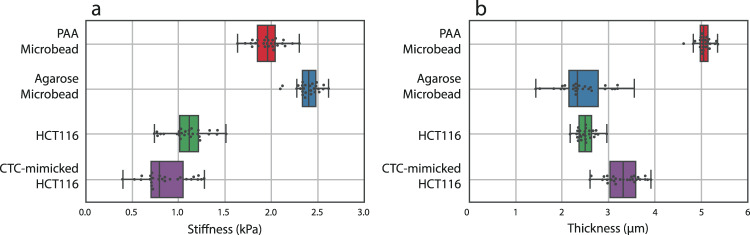
Stiffness and thickness measurements for reference microbeads, HCT116 cells, and CTC-mimicking HCT116 cells. **a** Stiffness and **b** thickness measurements represented as measured average values and standard deviations. The sample sizes for the experiment groups were *n* = 50, *n* = 50, *n* = 35, and *n* = 25 across 3 independent experiments for PAA microbeads, agarose microbeads, epithelial HCT116 cells, and TGF-β treated HCT116 cells (CTC-mimicking HCT116 cells), respectively. CTC-mimicking cells had a lower stiffness value compared to their epithelial counterparts. One-way ANOVA comparison yielded $P=2.20{{\mbox{e}}}-7$ ($F{{{{{\boldsymbol{=}}}}}}36.64,\,{\eta }^{2}=0.186$) for (**a**) and $P=1.25{{\mbox{e}}}-6$ ($F=45.71,\,{\eta }^{2}=0.172$) for (**b**). Box plot centers show median, box bounds show interquartile range and whiskers show 10th to 90th percentile.

Acousto-holographic measurements of PAA and agarose microbeads with sample sizes of 50 allowed us to characterize the particle-to-particle stiffness (Fig. 3a) and thickness (Fig. 3b) variations. For the measured ensemble of PAA and agarose microbeads, we find the stiffness as $1.94\pm 0.17$ kPa and $2.43\pm 0.11$ kPa and the thickness as $31.08\,\pm \,10.24$ µm and $34.68\,\pm \,13.45$ µm. These are in good agreement with the values reported in the literature^37,39,40^. These results validate the use of our acousto-holographic system for stiffness measurements (see Supplementary Table 1 for results demonstrating the repeatability of our measurements and stiffness values obtained in various media).

Finally, we used the determination of coefficient ${R}^{2}$ (see “Methods” and the Supplement) to compare the stiffness distributions obtained from acousto-holographic measurements, AFM measurements, and finite element analysis performed in COMSOL Multiphysics® (see Supplementary Text, Supplementary Figs. S13–S16, and Supplementary Tables 2 and 3 for details). We first calculated ${R}^{2}$ by comparing the histograms of bead stiffness distributions obtained from the acousto-holographic measurements with those obtained from COMSOL simulations, which resulted in ${R}^{2}=0.91\pm 0.02$, ${R}^{2}=0.89\pm 0.04$, and ${R}^{2}=0.88\pm 0.05$, respectively, for PS, PAA, and agarose microbeads, implying a good correlation between the measurements and simulations (Supplementary Table 2). ${R}^{2}$ values for PAA and agarose are slightly smaller because of slight variations in size, shapes, and refractive indices of particles used in simulations and experiments. Next, we compared the stiffness distributions obtained from acousto-holographic and AFM measurements of PAA and agarose microbeads. We found the correlation coefficients ${R}^{2}=0.90\pm 0.03$ and ${R}^{2}=0.93\pm 0.03$, respectively, for PAA and agarose, implying a very good correlation between our AFM and acousto-holographic measurements (Supplementary Table 3). The spatial resolution of the imaging system is determined to be in the range of 644 nm and 660 nm for lateral resolution and ~45 nm for axial resolution. The stiffness measurement resolution is determined to be ~0.25 Pa (See Supplementary Information for a detailed discussion of resolution).

### Stiffness maps of TGF-β treated and untreated epithelial HCT116 Cells

**Fig. 4:**
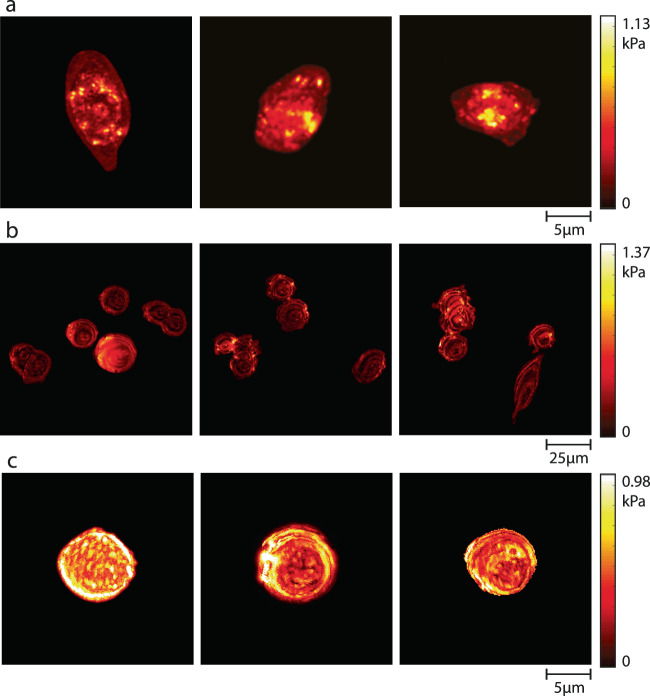
Acousto-holographic reconstruction provides high-resolution mapping of stiffness distribution in single and multiple cells. Panels **a** and **b** present, respectively, the stiffness distributions of single and multiple endothelial HCT116 cells obtained using acousto-holographic system. Slight difference in the stiffness distributions of cells in **a** and **b** may be attributed to cell-to-cell stiffness variations and interference effects. **c** Stiffness map of CTC-mimicking HCT116 cells that lose their adherent properties to metastasize. Cell stiffness is higher due to the stiffer and more regular distribution of actin filaments in HCT116 cells compared to CTC-mimicking HCT116 cells. For each sample, the results were obtained over three independent experiments.

We then reconstructed the stiffness and thickness maps of 35 epithelial HCT116 (Human Colorectal Carcinoma) cells (Figs. 3 and 4a, b) using the acousto-holographic system. The average stiffness of HCT116 cells is estimated as $1.08\pm 0.14$ kPa. Stiffness distributions of individual cells in the multiple cell environment slightly differ from those when the cells from the same cell type are measured individually, as seen in Fig. 4a, b. In addition to cell-to-cell stiffness variation, we attribute such differences to interference effects in multiple cell environment, in particular when cells are closely spaced, and to the associated noise in resolving individual cells in image processing. Image qualities and the resolution of cell stiffness distributions of individual cells in a multiple cell environment can be further improved by using better imaging systems and filtering. To confirm the validity of these measurements, we performed AFM measurements on a new set of 35 epithelial HCT116 cells which revealed stiffness value of $1.16\pm 0.22$ kPa, which is in good agreement with the results obtained from our acousto-holographic system. Finally, we calculated the similarity of the stiffness distributions obtained using AFM and acousto-holographic measurement results by comparing two-dimensional stiffness maps and associated stiffness histograms. From the 1225 calculated ${R}^{2}$ values, we obtained ${R}^{2}=0.82\pm 0.12$, implying a high level of similarity between stiffness distributions. Deviation from ${R}^{2}=1$ is attributed to cell-to-cell stiffness variation and noises in measurements and signal processing during reconstruction. Furthermore, AFM results are generally dependent on the type of the tip used and the sample preparation procedure. These shortcomings of the AFM method render an accurate pixel-by-pixel validation unfeasible through comparison with AFM results.

A significant advantage of our system over AFM for stiffness measurements is that it allows the holographic reconstruction of whole-cell stiffness maps for individual (Fig. 4a) and multiple cells (Fig. 4b) in the same environment in much shorter measurement times. Each stiffness map shown in Fig. 4 are reconstructed in ~10 s (i.e., 1 s acquisition and ~9 s processing time) using 1000 interferograms acquired at a spatial resolution of 1920 × 1216 (i.e., total number of pixels is 2,334,720). AFM studies that measure cell stiffness generally report a sampling rate less than 8 points/s^5,41,42^. Acquiring the same number of measurement points (i.e., 1920 × 1216 points) on a cell with an AFM will require ~3.8 days with a sampling rate of 7 points/s. This does not only limit the number of cells that can be analyzed in one day but also extend well beyond the measurement time relevant to cellular activities (~1–10 min)^43,44^. Limiting the number of acquired points, on the other hand, leads to significantly decreased resolution in whole-cell stiffness maps (see Supplementary Information for reconstructed stiffness map using an AFM). This renders reconstructing whole-cell stiffness maps using AFM unfeasible despite its high spatial resolution because the cell may lose its viability or undergo multiple morphological changes during this long measurement time. Cell lifetime and limited duration of cellular activities and processes require faster and high-resolution reconstruction of whole cell stiffness maps^45^. Recent progress in AFM technologies has led to high-speed scanning, significantly reducing the acquisition time. However, their use is mostly limited to rigid microbial cells or isolated molecules adsorbed on a substrate and to smaller scan size, typically less than one micrometer^39^. These combined with the special controlled environment and technical expertise needed to operate an AFM and the complex tip-sample interaction^46^ which may damage the cell or lead to accumulation of cell membrane material on the tip^47^ requiring frequent replacement of the costly AFM tip, limit the clinical and widespread use of AFM techniques. Our acousto-holographic system with its simplicity, speed, cost effectiveness, high-throughput, ability to measure whole-cell stiffness maps of single or multiple cells, and ease of use may open the way for clinical and widespread use of cell-stiffness measurement for early detection of diseases and monitoring their progression.

Next, we studied the effect of TGF-β (transforming growth factor-beta) treatment on cell stiffness maps. TGF-β is a multifunctional cytokine that is commonly used for inducing the epithelial–mesenchymal transformation (EMT) in various epithelial cell lines^48^. During this process, the epithelial characteristics deteriorate, and the cells acquire a migratory behavior. Deterioration of the epithelial characteristics includes dissolution of cell-cell junctions, adherent junctions, desmosomes, and loss of epithelial cell polarity. As a result, cells acquire a mesenchymal phenotype, characterized by actin reorganization and stress fiber formation, migration, and invasion. TGF-β signaling in the body is one of the main precursors of metastasis and is shown to increase the formation of circulating tumor cells (CTCs)^49^. Although the signaling pathways of the EMT have been extensively studied, the effects of the EMT on the mechanical behavior of individual cells are hindered due to the shortcomings of existing techniques. Studies have been mostly performed using AFM and the results of these studies are naturally affected by all the previously mentioned pros and cons of AFM. However, these studies revealed reduction in the elasticity modulus of hepatocarcinoma cells due to the metastatic effect induced by TGF-β treatment^50^; an increase up to 71% in the stiffness of cell monolayers (i.e., stiffer junctional regions and softer body parts) in proximal kidney tubule epithelial cells (NRK52E) treated by TGF-β due to intracellular F-actin distribution^51^; significant increase in the stiffness of the alveolar epithelial cells after TGF-β induced EMT^52^; and reduced cell stiffness in murine mammary gland epithelial cells (NMuMG) undergoing EMT induced by TGF-β^53^. New techniques, such as our acousto-holographic reconstruction of cell stiffness maps, that address the shortcomings of AFM and other existing methods will accelarate basic research and clinical studies, help to fully understand the mechanisms underlying EMT, and investigate the effects of TGF-β and its inhibition which may lead to development of novel therapies. Therefore, here we test the performance of our acousto-holographic technique for discriminating between TGF-β treated and untreated HCT116 cells.

We obtained CTC-mimicking HCT116 cells by treating HCT116 cells with TGF-β and reconstructed their stiffness maps using our acousto-holographic system after a 48 h incubation period. The stiffness maps (Fig. 4c) revealed that CTC-mimicking HCT116 cells (i.e., HCT116 cells treated with TGF-β) had a stiffness of $0.88\pm 0.16$ kPa (Fig. 3), which is smaller than $1.08\pm 0.11$ kPa obtained for the untreated HCT116 cells. A comparison of the stiffness histograms obtained from the reconstructed 2D maps of 35 HCT116 cells with those of 25 TGF-β treated HCT116 cells measured using the acousto-holographic system yield ${R}^{2}=0.42\pm 0.24$ (i.e., a total of 875 ${R}^{2}$ values were calculated). This implies a strong dissimilarity between the measured stiffness values of HCT116 and TGF-β treated HCT116 cells. Thus, we conclude that HCT116 cells have a stiffer profile than the TGF-β treated HCT116 cells and that our acousto-holographic system can confidently identify the stiffness difference between these two different types of cells. This result is expected due to the stiffer and more regular distribution of actin filaments in HCT116 cells than those in CTC-mimicking HCT116 cells. This change in stiffness induced by TGF-β treatment can be explained as follows. As the cells undergo transition to a malignant state, their cytoskeletal structures change from an organized network to an irregular network, which reduces the cell stiffness. In the malignant states, the cells usually have a smaller number of tensile fibers; residual microfilament bundles are irregular, and maturation of focal adhesions is impaired. As a result, singular tumor cells are softer than benign and healthy ones^54^. This is often associated with the changes in cell mechanics of the cancer cells to metastasize.

### TGF-β induced epithelial to mesenchymal transition of HCT116 Cells

**Fig. 5:**
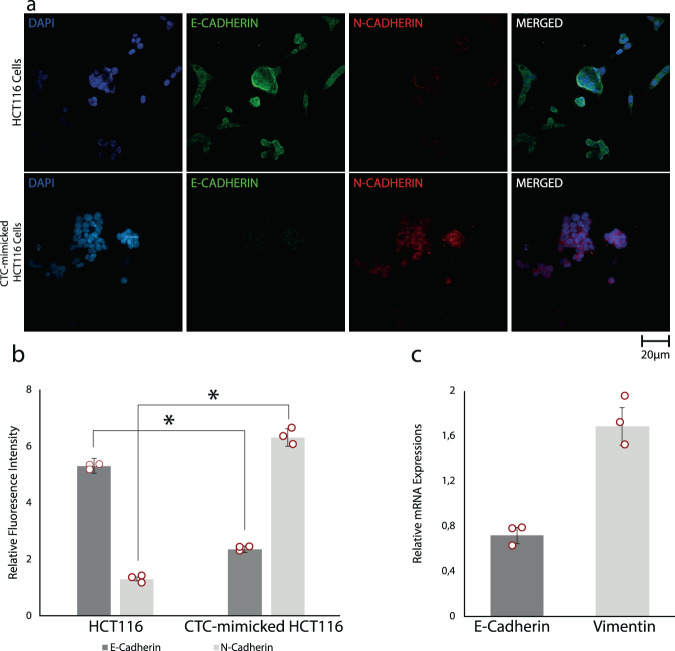
Validation of mesenchymal character induced by TGF-β treatment. **a** Fluorescence immunocytochemistry staining for DAPI (blue), E-cadherin (green), and N-cadherin (red) on HCT116 and CTC-mimicking HCT116 cells, respectively. E-cadherin is down regulated whereas N-cadherin is upregulated in CTC-mimicking cells. Scale bar 20 µm. **b** Bar graphs demonstrating the intensity of expressed E-cadherin and N-cadherin. Quantification of fluorescence intensities from arbitrary images of each condition was done with ImageJ software (NIH, Bethesda, MD, USA). **c** E-cadherin and vimentin mRNA expression relative to housekeeping gene *GAPDH* by ${2}^{-\Delta \Delta {{{{{\rm{Ct}}}}}}}$ value calculation. E-cadherin expression was decreased 0.7-times and vimentin expression was increased 1.7-times after TGF-β treatment for 48 h. All data in **b** and **c** are expressed as mean ± S.D. (standard deviation) for data collected in *n* = 3 over three independent experiments and star (*) indicates *p* < 0.05 obtained via unpaired Student’s t test.

Finally, we performed fluorescence immunocytochemistry staining experiments (Fig. 5) to confirm the mesenchymal character induced by TGF-β treatment and support our arguments on the reasons for the difference in the stiffness of HCT116 and CTC-mimicking HCT116 cells. The transition from epithelial to mesenchymal form plays an important role in the formation of CTC cells from epithelial cancer cells, increasing the invasion ability of tumor cells and enabling their survival in the peripheral system. Epithelial-to-mesenchymal transition process consists of many molecular and cellular changes such as down-regulation of epithelial proteins (e.g., E-cadherin) and up-regulation of mesenchymal proteins (e.g., N-cadherin). This was observed in our experiments as decreased E-cadherin and increased N-cadherin expressions for CTC-mimicking HCT116 (Fig. 5a, b), clarifying epithelial–mesenchymal transition process and the formation of CTC-mimicking HCT116 cells via TGF-β treatment. We further confirmed this with quantitative real-time polymerase chain reaction (qRT-PCR) of E-cadherin and vimentin mRNA expressions after TGF-β treatment. Vimentin is linked to increased tumor growth and invasiveness and considered to be a major biomarker of EMT^55^. A recent study has also revealed that vimentin plays an active role in controlling and reorganizing cellular architecture towards a migratory and invasive phenotype^56^. Our analysis showed 0.7-fold decrease in E-cadherin and 1.7-fold increase in the vimentin expressions after TGF-β treatment for 48 h. These results prove and confirm that the epithelial–mesenchymal transition was realized, and our acousto-holographic cell stiffness mapping technique can detect this transition and differentiate between epithelial and mesenchymal forms of the cell. Our results also indicate that the difference in stiffness of the cells is caused by the difference in their cellular stresses which deform extracellular-matrix (ECM) structure through the changes in the cytoskeleton^57^.

## Discussion

In this study, we developed an acousto-holographic imaging system for the observation of acoustic bulk wave compression of cells that allowed us to perform untethered measurement of cell stiffness distribution at sub-micron resolution without compromising cells’ viability. This then made it possible to differentiate between epithelial and mesenchymal forms of cells based on their stiffness distributions. The technique provides high-resolution whole-cell stiffness maps of single and multiple cells with a speed and resolution beyond what is achievable with AFM- and OT-based methods. With further improvements in the detection and imaging components, the technique would be capable of measuring subcellular stiffness distribution. Due to its simplicity, the system developed here can be integrated to an incubator to continuously monitor changes in cell stiffness under various biological, physical, and chemical conditions and perturbations. We think the system and techniques developed here will become a valuable tool for studying alterations in the mechanical properties of individual cells and multiple cells in the same environment and may lead to the development of rapid biomechanical assays that use cell stiffness maps as biomarkers of diseases (e.g., cancer, neurodegenerative diseases, etc.) and disease pathogenesis and progression, as well as drug efficacy tests.

## Methods

### Fluidic chamber

The fluidic chamber was fabricated on a microscope cover glass slide (0.13–0.17 mm thickness) using polydimethylsiloxane (PDMS) Sylgard 184 elastomer kit. The PDMS base and curing agent were mixed in a weight ratio of 10:1 and kept in the vacuum to remove air bubbles for 30 min. After degassing, a PZT transducer (SMBA25W7T05PV, Steiner & Martins Inc.) was placed on the polymer and PDMS was cured in the oven at 65 °C for 3 h. A 12 mm × 12 mm fluidic chamber was created at the end of the piezoelectric transducer placed in the PDMS structure. The piezoelectric transducer has a resonant frequency of 1 kHz with a displacement of 0.05 mm at an applied peak-to-peak voltage of 24 V. PDMS structure was irreversibly bonded to the microscope glass slide using high power Oxygen plasma at 400 mTorr pressure for 2 min. The fluidic chamber was sterilized by sonicating in ethanol for 5 min.

### Cell culture

The human colorectal adenocarcinoma (HCT116, ATCC® CCL-247) was obtained from the American Type Culture Collection-ATCC (Manassas, VA, USA). These cells were propagated and maintained in McCoy’s 5A (Cegrogen, Germany) cell culture media supplemented with 10% fetal bovine serum (Cegrogen, Germany), 1% penicillin/streptomycin (Cegrogen, Germany) and 1% L-glutamine (Cegrogen, Germany) in humidified incubator at 37 °C with 5% CO_2_. HCT116 cells were then treated with the transforming growth factor beta (TGF-β, R&D Systems, 240-B) (10 ng/mL) to stimulate CTC-mimicking cells displaying a mesenchymal phenotype. Acousto-holographic measurements of HCT116 and TGF-β treated HCT116 (CTC-mimicking HCT116) cells were conducted in a DMEM/F12 (Invitrogen) solution which had a refractive index value of 1.337^58^. Measurements of PS, PAA, and agarose microbeads were performed in deionized (DI) water solution at 24 °C with a 532 nm continuous-wave (CW) laser. At this working condition, the refractive index of the DI water is 1.334^59^. The refractive index of 8% PAA microbeads are reported as 1.349 and 0.04% agarose microbeads as 1.3329 at 23 °C measured with a light source of 589 nm^60^. Refractive index of PS microbeads are reported as 1.5915 at 589 nm at 25 °C^61^. For comparison purposes, we also performed stiffness measurements of these microbeads in DI water, DMEM/F12, and glycerol (see Supplementary Figs. S6 and S7, and Supplementary Table 1). Refractive index of glycerol is reported as 1.4722 at 589 nm at 25 °C^62^.

### Experimental setup

A phase-shifting inline Mach–Zehnder interferometer was used in our holographic imaging system (see Fig. 1). We employed a 527 nm, 10 mW He-Ne laser as a coherent light source. The laser beam is first expanded (Newport T81-10X beam expander) and then input to the first beamsplitter whose outputs are sent to the two paths of the interferometer. Phase shifting is performed by varying the length of one path of the interferometer with a high-frequency piezo actuator (New Focus, Picomotor 8302) operated at 1000 steps per second. Each step of the piezo actuator is 30 nm corresponding to $\,{{{{{\rm{\pi }}}}}}$/10 rad of phase change. The phase is updated every 50 ms. The microfluidic chamber containing the cell culture (or reference microbeads) is placed in the other path of the interferometer and a PZT transducer (SMBA25W7T05PV, Steiner & Martins Inc.) provides an acoustic stimulation with a frequency of about 1 kHz. Light fields after the phase-shifting unit and the microfluidic chamber are collected by microscope objective lenses (Newport M-10X, 10x magnification, 0.25 numerical aperture, 16.5 mm focal length, and 7.5 mm clear aperture) and recombined at the second beamsplitter of the interferometer. A CMOS camera (ZEISS, AxioCam 702 mono) synced with the piezo actuator’s motion at a frequency of $\sim 1\,{{\mbox{kHz}}}+\varDelta f$ Hz records the interference patterns at one of the outputs of the beamsplitter. At each phase step 50 interferograms are acquired. We repeat the process for 20 steps and acquire a total of 1000 interferograms within 1 s. A code developed in-house is used to process the acquired interferograms for holographic reconstruction and stiffness measurement (i.e., processing time is ~9 s on a system with AMD Ryzen Threadripper 1950X processor and 32GB RAM). Acoustic excitation and manipulation may impact the viability of in vitro cell cultures through thermal or cavitation effects^63^. Thermal effects are the result of the absorption of the acoustic energy by the cellular body, and they are significant at higher acoustic frequencies (e.g., ~1–10 MHz). Cavitation effects, on the other hand, are more dominant at lower frequencies. It has been shown that low power (<2 Watts) cavitation applications generally have a positive effect on the viability of in vitro cell cultures^64^. The acoustic signal used in our experiments for cell stiffness measurements had a power of 0.05 W at 1 kHz. Therefore, we expect negligible thermal and cavitation effects that may affect the viability of cells in a negative way, and our system can be safely considered as minimally invasive. We did not observe any difference in cell viability during prolonged measurements.

### Reconstruction of stiffness maps

We collect 50 interferograms at each of the 20 phase steps where each step corresponds to a phase shift of $\pi /10$. After the image acquisition is completed, we use a wavelet transform based phase matching algorithm^65^ to determine how many frames should be shifted so that the frames of two consecutive interferograms obtained at each phase step are well-matched. The algorithm gives a measure of how likely a given interferogram is the phase-shifted pair of another interferogram and thus allows us to match the frames of consecutive interferograms. We repeat this process for each phase step and then use a least-squares minimization-based reconstruction technique^66^ to minimize the error induced by the remaining mismatch in the phase difference between frames. Reconstructing each set for increasing phase shifts gives us a continuous video of the vibration pattern generated on the cell surface. We then create 50 bins each of which contains one of the 50 interferograms obtained at one phase step. Each bin thus contains interferograms collected within 1/50-th portion of a single period of the vibration. We place interferograms obtained at the 20 phase steps into the same bins, resulting into 50 bins each with 20 interferograms. After the binning is completed, we first filter each interferogram to reduce noise and then apply a phase retrieval algorithm^67^ (see Supplement for details) at each bin to place the interferograms according to the increasing phase-shift. We also do phase unwrapping using the preconditioned conjugate gradient (PCG) algorithm^68,69^. This algorithm is based on first computing the discrete cosine transform of the raw interferogram, then solving the discrete Poisson equation, and finally computing the inverse cosine transform. After phase unwrapping, we perform phase reconstruction and extract the thickness (see Supplement for details). Using the thickness information, we estimate the change in the thickness within one period of acoustic simulation for each point on the sample and obtain a thickness map. Subsequently, for each point, we iteratively compare thickness map with the thickness data obtained from the response of a two-dimensional linear-elastic membrane model to determine the stiffness coefficient that gives the best fit. A detailed description of the image acquisition, phase retrieval, and stiffness estimation along with a schematic representation is given in the Supplementary Material.

### Filtering for noise reduction

We used a BM3D based filtering^70^ to reduce the noise content of the reconstructed thickness maps. Filter is applied onto individual interferograms and the thickness maps were reconstructed from the filtered set of interferograms. We validated the noise reduction method by reconstructing the thickness maps of a reference slide (Malvern, PVS 5113) and reference PS beads (Thermo Scientific, 4K100). These reference objects have pre-defined shapes and are suitable for measuring the error rates of the obtained thickness maps. We note that ground truth thickness information of the cell cultures was not available to make a comparison with the reconstructed thickness. Therefore, we validated the effectiveness of the noise reduction method, by comparing the estimated thickness information obtained from measurements with and without the medium flow. After the observation, the medium flow is started again so as not to disturb the dynamic culture. We note that this method compensates for transitory deformations in the fringe patterns induced by the fluid flow in dynamic cell cultures.

### Statistical analysis

We used the coefficient of determination

1 $${R}^{2}=1-\frac{\sum {\left({y}_{i,{{\mbox{observed}}}}-{y}_{i,{{\mbox{calculated}}}}\right)}^{2}}{\sum {\left({y}_{i,{{\mbox{observed}}}}-\bar{y}\right)}^{2}}$$

with $\bar{y}={N}^{-1}\sum {y}_{i,{{\mbox{observed}}}}$ to quantify how well the estimated and calculated phase distributions agree. The sum operation was carried over the histogram of two-dimensional data obtained from the calculated phase distribution and the cellular vibration observations. This method is commonly used for analyzing the similarity between two distributions^71^. We used ${R}^{2}$ to assess similarity and dissimilarity of stiffness maps obtained from AFM, acousto-holographic system, and COMSOL simulations (see Supplementary file for details) and to assess the stiffness dissimilarity between epithelial HCT116 cells and TGF-${{{{{\rm{\beta }}}}}}$ treated HCT116 cells measured using the acousto-holographic system. Statistical analyses were performed using MATLAB. We performed one-way ANOVA for comparisons between multiple groups with the null hypothesis that the mean values are the same. To demonstrate the repeatability of the acousto-holographic method, we performed ten measurements on each microbead and cell. The sample sizes used in the experiments were 50 for PAA, agarose, and PS microbeads, and 35 and 25 for epithelial HCT116 cells and TGF-β treated HCT116 cells, respectively. The variances of stiffness measurements were found as 0.11, 0.12, 0.14, 0.21, and 0.32 kPa, respectively, for PS microbeads, PAA microbeads, agarose microbeads, epithelial HCT116 cells, and TGF-β treated HCT116 cells (see also the Supplementary Text).

### Reporting summary

Further information on research design is available in the Nature Portfolio Reporting Summary linked to this article.

## Supplementary information

Supplementary Information

Reporting Summary

## Data Availability

The depth map data used in this study are available in the Zenodo database under 10.5281/zenodo.7197107. Raw holographic recordings were not made public due to their large size (>1TB) but are available upon request from the corresponding author.

## Code availability

The code that was used to obtain the results presented in this paper are available through the link https://github.com/rvarol/acousto-holographic-stiffness-measurement.
