# Supplementary material for: Acousto-holographic reconstruction of whole-cell stiffness maps
Source: Nat Commun. 2022 Nov 29;13:7351. doi: 10.1038/s41467-022-35075-x (PMC9709086; doi:10.1038/s41467-022-35075-x)
Supplement: Supplementary file 1 — Supplementary Information [file 41467_2022_35075_MOESM1_ESM.pdf]

# Acousto-Holographic Reconstruction of Whole-Cell Stiffness Maps

Rahmetullah Varol[1,†], Zeynep Karavelioglu[2,††], Sevde Omeroglu[3], Gizem Aydemir[1,††], Aslihan Karadag[4], Hanife Ecenur Meco[4], Ali Anil Demircali[1,†††], Abdurrahim Yilmaz[1], Gizem Calibasi Kocal[4], Gulsum Gencoglan[5], Muhammed Enes Oruc[3], Gokhan Bora Esmer[6], Yasemin Basbinar[4], Sahin Kaya Ozdemir[7], Huseyin Uvet[1*]

[1]Mechatronics Engineering Department, Yildiz Technical University, Istanbul 34349, Turkey

[2]Bioengineering Department, Yildiz Technical University, Istanbul 34220, Turkey

[3]Chemical Engineering Department, Gebze Technical University, Kocaeli 41400, Turkey

[4]Oncology Institute, Dokuz Eylul University, Izmir 35330, Turkey

[5]Department of Dermatology, Istinye University, İstanbul 34010, Turkey

[6]Electrical and Electronics Engineering Department, Marmara University, Istanbul 34722, Turkey

[7]The Pennsylvania State University, Department of Engineering Science and Mechanics, University Park, PA 16802, USA

*Corresponding author: Huseyin Uvet (huvet@yildiz.edu.tr)

[†]Current address: Universität der Bundeswehr München, Munich 85577, Germany

[††]Current address: Eidgenössische Technische Hochschule Zürich, Zürich 8092, Switzerland

[†††]Current address: Hamlyn Centre, Imperial College London, London SW7 2AZ, UK

## *S.1. Supplementary Text*

## *S.1.1 Hertzian Model*

Throughout an acoustic field, a non-zero time-averaged stress is created by non-linear terms in the Navier-Stokes equations. This stress is discontinuous across the boundary of a cell and its effect can be approximated in the non-viscous regime as a stress difference[1]:

$$\Delta\Pi = \Pi_{\mathrm{i}} - \Pi_{\mathrm{o}} \quad \text{(S1)}$$

where $\Pi_{\mathrm{i}}$ and $\Pi_{\mathrm{o}}$ are the j on the inside and the outside of the membrane, respectively, given to second order as[2]

$$\Pi_{\mathrm{jk}} = -\langle P - P_o\rangle\delta_{jk} - \rho_0\langle v_j v_k\rangle \quad \text{(S2)}$$

where $\delta_{jk}$ is the Kronecker delta, $\langle \dots \rangle$ represents the time average. The second term on the right-hand side is the Reynolds stress, representing the time-averaged transport of the momentum density $\rho_0 v_j$ with velocity $v_k$ in the direction of the surface normal. The time averaged mean Eulerian excess pressure is given by[3]

$$\langle P - P_o\rangle = \frac{1}{2\rho_0 c_0^2}\langle p^2\rangle - \frac{\rho_0}{2}\langle v^2\rangle \quad \text{(S3)}$$

with $\rho_0$, $c_0$, $p$, and $v$ denoting the quiescent fluid density, ambient speed of sound, first order acoustic pressure, and first order acoustic velocity, respectively. Through this formulation, elastic modulus $E$ of the membrane is calculated through the linear relation

$$E_{jk} = \Pi_{\mathrm{jk}}/\epsilon_{jk} \quad \text{(S4)}$$

where $\epsilon$ is the strain. In our acousto-holographic system, the strain $\epsilon$ is calculated from the measured displacement $d$ using

$$\epsilon = d/\phi_0 \quad \text{(S5)}$$

where $d = \phi - \phi_0$ with $\phi_0$ denoting the initial thickness of the sample and $\phi$ corresponding to the thickness at the instant of the acoustic pressure. These definitions will become clearer in the following sections.

## *S.1.2 Processing of acquired interferograms*

As explained in the Methods section of the Main text, the acquired 50 interferograms at 20 different phase steps are first processed using a wavelet transform based phase matching algorithm and stored in 50 bins, each containing one of the 50 interferograms obtained at one phase step (50 bins each with 20 interferograms), then filtered for noise reduction, and finally processed using phase retrieval and phase unwrapping algorithms. The resulting interferograms are then processed to extract the displacement $d$ which is used in Eq. (S5) to calculate strain $\epsilon$, which is subsequently used to calculate the elastic modulus $\delta$ using Eq. (S4). Below is the outline of the mathematical basis of displacement $d$ measurement from the interferograms.

The intensity measured at the $j$-th pixel ($j = 1,2,\dots,N$) in each image of the $i$-th phase-shifted interferogram ($i = 1,2,\dots,M$) can be expressed as[4]

$$I_{ij} = A_{ij} + B_{ij}\cos(\theta_j + \delta_i) \quad \text{(S6)}$$

where $A_{ij}$ and $B_{ij}$ are respectively the background intensity and the modulation amplitude, $\theta_j$ are the angular phase, and $\delta_i$ are the phase-shift amount at each frame. We assume that $A_{ij}$ and $B_{ij}$ do not have intra-frame variation. This is a reasonable assumption under stable imaging conditions. Then, the expression given in Eq. (S6) can be written as

$$I_{ij}^t = a_j + b_j\cos(\delta_i) + c_j\sin(\delta_i) \quad \text{(S7)}$$

where $a_j = A_{ij}$, $b_j = B_{ij}\cos(\theta_j)$, and $c_j = -B_{ij}\sin(\theta_j)$. Using $M$ images and $N$ pixels we use an over-determined least-squares method to solve for the unknown variables. The least-squares error $S_j$ is given by

$$S_j = \sum_{i=1}^{M}\left(I_{ij}^t - I_{ij}\right)^2 = \sum_{i=1}^{M}\left(a_j + b_j\cos(\delta_i) + c_j\sin(\delta_i) - I_{ij}\right)^2 \quad \text{(S8)}$$

where $I_{ij}$ is the experimentally measured intensity of the interferogram. Least-squares criteria are expressed by $\partial S_j/\partial a_j = 0$, $\partial S_j/\partial b_j = 0$, and $\partial S_j/\partial c_j = 0$ which yield $X_j = A^{-1}/B_j$ with

$$A = \begin{bmatrix} M & \sum_{i=1}^{M}\cos(\delta_i) & \sum_{i=1}^{M}\sin(\delta_i) \\ \sum_{i=1}^{M}\cos(\delta_i) & \sum_{i=1}^{M}\cos^2(\delta_i) & \sum_{i=1}^{M}\cos(\delta_i)\sin(\delta_i) \\ \sum_{i=1}^{M}\sin(\delta_i) & \sum_{i=1}^{M}\cos(\delta_i)\sin(\delta_i) & \sum_{i=1}^{M}\sin^2(\delta_i) \end{bmatrix} \quad \text{(S9)}$$

$$\{X_j\} = \{a_j \quad b_j \quad c_j\}^T \quad \text{(S10)}$$

$$\{B_j\} = \{\sum_{i=1}^{M} I_{ij} \quad \sum_{i=1}^{M} I_{ij}\cos(\delta_i) \quad \sum_{i=1}^{M} I_{ij}\} \quad \text{(S11)}$$

Using Eqs. (S9) - (S11), we estimate the unknowns $a_j$, $b_j$, and $c_j$ and then use the estimated values to calculate the thickness $\phi_j$ as:

$$\phi_j = \tan^{-1}(-c_j/b_j) \quad \text{(S12)}$$

We then find the displacement (i.e., the change in membrane thickness in response to acoustic pressure) using $d = \Delta\phi = \phi - \phi_0$ in response to applied acoustic pressure. Here, $\phi_0$ is the initial thickness of the sample and $\phi$ is the thickness at a specific instant of the acoustic pressure. Plugging the calculated $d$ in Eq. (S5) gives the strain $\epsilon$ which is then used in Eq. (S4) to calculate the elastic modulus $E$.

### *S.1.3 Finite element studies*

To further validate the stiffness results obtained from microbead experiments and to compare the distribution of stiffness over the microbead, we have conducted finite element analysis (FEA) studies using COMSOL Multiphysics®. A schematic description of the analysis and comparison process is shown Figure S12. Within the context of this analysis, we consider the materials as a homogeneous viscoelastic material. Under this assumption, a continuum model description of microbead deformation can be applied and implemented in COMSOL.

Three different physics interfaces are used to simulate the model. The continuum mechanics of the microbeads and the acoustic pressure are modeled using the "Solid Mechanics" and the "Pressure Acoustics" module, respectively. These modules are coupled using the "Acoustic-Structure Interaction" coupling module. For resolving the optical interference patterns, the "Wave Optics" module was used. The PDMS microwell chip is defined as a rectangular PDMS block with 24 mm width, 60 mm length and 3 mm depth. The well itself is defined as a rectangular block of 20 mm width, 20 mm length and 3 mm thickness placed within the PDMS with a custom material named

“Medium”. Simulations were performed for PS, PAA, and agarose microbeads (i.e., material properties are given in Supplementary Table 2). The microbeads are defined as spherical particles at the middle of the well attached to the bottom. The PZT transducer was defined as a circular block of PZT material with a 15 mm diameter and a 2 mm thickness. The horizontal and vertical pathways through which laser beams travel were modeled as a rectangular block running parallel and orthogonal to the length of the PDMS chip, respectively. A quartz beam splitter was defined as the isosceles right triangular block with 10 mm side length and 10 mm thickness. A transition boundary condition between these triangular blocks was defined to simulate the splitting of incident laser beams. An acoustic-structure boundary was defined between the sphere and the medium. The acoustic signal input to the system is defined with a spherical wave radiation source defined at bottom-centers of one of the side walls. A gaussian beam with a 1 mm radius was defined to travel across the horizontal and vertical optical pathways. The interference pattern was measured by plotting the electric field intensity at the bottom surface of the vertical optical pathway. A two-step study was conducted with the first step being a “Transient Analysis” involving the “Solid Mechanics” physics and the “Pressure Acoustics” physics. The interval of solution was defined between 0 s and 1e-3 s with a 1e-5 s step size. For each transient solution, a secondary “Frequency Analysis” study involving the “Wave Optics” module was conducted. The solution was defined at the wavelength of 521 nm. Furthermore, for each “Frequency Analysis” a parametric sweep was defined for the phase delay of the horizontal optical beam (reference beam) starting from 0 up to $2\pi$ with $2\pi/5$ intervals. Through solving this sweep for each time interval, the phase patterns shown in Figure S12 (b) were obtained. These patterns were then reconstructed using MATLAB to obtain the thickness maps shown in Figure S12 (c). We then applied the methodology described in Section S.1.1 to obtain the stiffness maps shown in Figure S12 (d).

## *S.1.4 Method validation using coefficient of determination*

Examples of stiffness maps obtained from our acousto-holographic measurements and the COMSOL simulations for PS, PAA, and agarose microbeads are shown in Figure S13. Examples of histograms of the stiffness values extracted from the 2D stiffness maps, which are reconstructed through AFM and acousto-holographic measurements, and COMSOL simulations are given in Figures S14, S15, and S16, respectively for PAA, agarose, and PS microbeads. AFM and acousto-holographic measurements were performed on different microbeads drawn from the same batch. Figure S17 shows exemplary stiffness histograms obtained from 2D stiffness maps measured for epithelial HCT116 cells using the acousto-holographic system and AFM, and stiffness histograms obtained for CTC-mimicking HCT116 cells using the 2D stiffness maps reconstructed using the acousto-holographic system. In COMSOL simulations we assumed the size of the microbeads as the average size of the microbead batches used in the experiments.

We compared these histograms using the coefficient of determination formula (explained in the Statistical Analysis)

$$R^2 = 1 - \frac{\sum\left(y_{i,\text{observed}} - y_{i,\text{calculated}}\right)^2}{\sum\left(y_{i,\text{observed}} - \bar{y}\right)^2} \tag{S13}$$

with $\bar{y} = N^{-1} \sum y_{i,\text{observed}}$ where $y_{i,\text{observed}}$ is the value at the $i - th$ bin in the histogram of experimentally obtained 2D map, and $y_{i,\text{calculated}}$ is the value at the $i - th$ bin in the histogram of 2D map obtained using FEA in COMSOL. This method is commonly used for analysing the similarity between two distributions[5]. The summations in (S13) run through all bins in the histograms. Using (S13), we obtained the coefficient of determination values as $R^2$=0.89, $R^2$=0.90, and $R^2$=0.94 for PAA, agarose, and PS microbeads, respectively, whose stiffness maps obtained from simulations are given in Figure S13.

Supplementary Table 2 presents the $R^2$ values obtained by comparing the stiffness histograms obtained from acousto-holographic measurements with those obtained from COMSOL simulations using a sample size of 50 PS, 50 PAA, and 50 agarose microbeads. $R^2$ values calculated are: $0.90 \leq R^2 \leq 0.92$, $0.87 \leq R^2 \leq 0.91$, and $0.86 \leq R^2 \leq 0.90$, respectively for PS, PAA, and agarose microbeads, with the overall statistics given as: $R^2 = 0.91 \mp 0.02$, $R^2 =$

$0.89 \mp 0.04$, and $R^2 = 0.88 \mp 0.05$, implying a good correlation between our measurements and simulations. The slightly lower correlation for PAA and agarose microbeads may be due to the difference in the actual shape of the microbeads used in the experiments and the perfectly spherical shape of the microbeads used in the simulations as well as small variations in the refractive indices. Furthermore, we have also compared the stiffness map resulting from COMSOL simulation and from AFM measurements for both PAA and agarose microbeads. This comparison yielded an $R^2$ value of 0.83 and 0.81 for PAA and agarose microbeads, respectively.

Supplementary Table 3 presents the $R^2$ values obtained by comparing the stiffness histograms obtained from acousto-holographic measurements with those obtained from AFM measurements on 50 PAA and 50 agarose microbeads. $R^2$ values calculated are: $0.85 \leq R^2 \leq 0.96$ and $0.85 \leq R^2 \leq 0.98$, respectively for PAA, and agarose microbeads, with the overall statistics given as: $R^2 = 0.90 \mp 0.03$ and $R^2 = 0.93 \mp 0.03$, implying a very good correlation between our AFM and acousto-holographic measurements. A summary of these results is depicted in the Supplementary Figure S18.

The boxplots in the Supplementary Figure S18 summarizes the $R^2$ values obtained by comparing the stiffness distributions (i.e., stiffness histograms) obtained from the 2D stiffness maps of PAA microbeads, agarose microbeads, and epithelial HCT116 cells which are experimentally reconstructed using acousto-holographic and AFM measurements. Figure S18 also includes the $R^2$ value that compares the results of acousto-holographic measurements for epithelial HCT116 and CTC-mimicking cells. We compared the stiffness histograms obtained from a total of 1225 two-dimensional stiffness maps (35 from AFM and 35 from acousto-holographic system) reconstructed using acousto-holographic and AFM measurements which yield $R^2 = 0.82 \mp 0.12$, implying that AFM and acousto-holographic measurements present a good correlation and agreement. Deviation from the ideality may be attributed to the cell-to-cell stiffness differences (i.e., a total of 70 cells were measured, 35 with AFM and 35 with acousto-holographic measurement system) as well as measurement and signal processing noises. Finally, we compared the stiffness histograms obtained for epithelial HCT116 cells and CTC-mimicking cells from 2D stiffness maps constructed from acousto-holographic measurements. In this comparison, we used 35 stiffness maps and associated histograms obtained for epithelial cells and 25 stiffness maps and associated histograms obtained for CTC-mimicking cells (i.e., a total of 875 two-dimensional maps

and associated histograms were compared). This comparison yielded $R^2 = 0.42 \mp 0.24$, implying a clear discrepancy between the histograms of stiffness maps of these cells as expected.

## *S.1.5. Determination of the spatial resolution*

In this section we summarize the studies that we have conducted in order to determine the spatial resolution and stiffness resolution of our system. Spatial resolution is further classified into lateral and axial resolutions. Lateral resolution is defined as the minimum distance between two point-objects of equal irradiance that produce two distinct reconstructed images. Axial resolution is defined as the minimum change in thickness that is required to obtain two distinct reconstructed images. Similarly, stiffness resolution is defined as the minimum change in stiffness that is required to obtain two distinct stiffness maps.

In digital holography, the discrete pixels on a CMOS or CCD array sample the continuous spatial distribution of an optically created hologram. The outputs of these pixels are then transformed to digitized signals and stored in an image processing system for numerical analysis. This matrix-based numerical processing that follows the digital sampling principle creates novel discretization effects that are not encountered in traditional holographic techniques. Digital holographic imaging systems are affected by at least the following three parameters that determine the lateral resolution[6]:

(1) the spacing between adjacent pixels of the CMOS,
(2) an averaging effect introduced by the finite size of these pixels,
(3) the finite extent of the camera face itself.

The main parameters that determine spatial resolution are pixel size and pixel number. Normally, the interferogram is captured through a rectangular array of pixels organized in $N$ rows and $M$ columns. In the case of a square image sensor $N = M$ holds. The distance between the centers of two consecutive pixels are called pixel pitch and denoted as $\Delta\xi$ and $\Delta\eta$ in the two orthogonal directions. For a square sensor with identical spacing in vertical and horizontal directions $\Delta\xi = \Delta\eta$. According to the Nyquist sampling theorem, a signal can be captured faithfully up to the following spatial frequencies[7]

$$f_{N\xi} = \frac{1}{2\Delta\xi} \text{ and } f_{N\eta} = \frac{1}{2\Delta\eta} \quad \text{(S14)}$$

However, typically the signal captured from the object is magnified through an objective lens. As such, the diffraction limits introduced by the lens must also be taken into account. In diffraction-limited systems, lateral resolution is typically defined by the Abbe's criterion[8]. This resolution is also called the two-point resolution as it gives the minimum distance between two point-like light sources that is required to resolve them. Previous studies[9,10] have shown that the minimum resolvable distance $\delta$ between two point sources is given by

$$\delta = 0.61\frac{\lambda d}{H} \quad \text{(S15)}$$

where $d$ is the distance between the image plane and the object plane and $H$ is the size of the image sensor. For our configuration with $\lambda = 527$ nm, $H = 7.1$ mm, and $d = 14.45$ mm, this formulation yields a spatial resolution of 654nm. In order to test and validate this theoretical calculation, we used 15$\mu$m PS microbeads (Sigma Aldrich, 74964) to determine the spatial dimension that can be measured with a single pixel. The reconstructed stiffness map of an exemplary microbead is shown in Supplementary Figure S19-a. The diameter of the microbead is measured to be equal to 46 pixels. Considering a standard deviation value of 0.2$\mu$m, it can be determined that a single pixel corresponds to a spatial dimension between 322nm and 330nm. Empirically, the two-point resolution can be approximated to be between 644nm and 660nm. A similar calculation is then conducted on an exemplary PS microbead with a 100$\mu$m diameter and the results are shown in Supplementary Figure S19-b. The thickness, diameter and average stiffness values of an ensemble of 50 PS microbeads is shown in Supplementary Figure S20. The diameter values measured as such are also in support of the spatial resolution calculated above. Furthermore, the cross-sectional stiffness measurements taken from the exemplary microbead is shown in Supplementary Figure S21.

The axial resolution on the other hand is limited by the smallest change in the thickness information which can be measured by our holographic imaging system. The thickness information is related to the phase measurement through Eq. (S12). Thus, it can be concluded that the accuracy with which we can measure the stiffness change at a given point is related to the accuracy with which we can measure change in phase. In order to determine the phase measurement accuracy, we analyzed the data obtained from PS microbeads. These microbeads have spherical shape, which allowed us to determine the expected change in thickness at different points on the imaging plane.

Thus, we were able to graph the change in phase as a function of change in thickness by measuring the phase information at the center of the microbead and comparing this value to the phase information measured at different points on the microbead. The real distance was calculated as a function of discrete pixel distances $\Delta x$ and $\Delta y$, respectively in the two orthogonal directions, as follows:

$$d = f(\Delta x, \Delta y) = \sqrt{(p\Delta x)^2 + (p\Delta y)^2} \tag{S16}$$

$$p = \frac{D_{\text{bead,real}}}{D_{\text{bead,pixel}}} \tag{S17}$$

where $D_{bead,real}$ is the real diameter of a PS microbead and $D_{bead,pixel}$ is the pixel count alongside the diameter of a given PS microbead. Furthermore, we calculate the expected change in thickness as a function of distance from the center as follows:

$$\Delta\phi_{\text{ex}} = \phi_c - \sqrt{\phi_c^2 - d^2} \tag{S18}$$

where $\phi_c$ denotes the thickness value measured at the center of the microbead. By measuring the phase change relative to the center of the microbead as a function of expected change in thickness we determined the following linear relation between them:

$$\Delta\phi = 0.0447\Delta\theta_{\text{e}} \tag{S19}$$

where $\theta_e$ is the 16-bit encoded phase information. From this, we infer the accuracy of our thickness measurement as $\sim 0.045\mu m$.

In order to translate the thickness measurement accuracy into stiffness measurement accuracy, we need to determine the minimum stiffness difference that would result in such thickness change under a given acoustic pressure and frequency.

The acoustic pressure value that is generated by our system can be determined using Eq. (S2). Assuming a linear elastic membrane model, the elasticity formula can be written as

$$E_{jk} = \frac{\Pi_{\text{jk}}}{\epsilon_{jk}} = \frac{\Pi_{\text{jk}}}{(\phi_0 + \Delta\phi)/\phi_0} \tag{S20}$$

where $\Pi_{\text{jk}}$ is the acoustic stress incident on the sample as described in Section S.1.1. Eq. (S20) relates the stiffness resolution of the system to the acoustic pressure and the initial thickness of the

measured point on the sample. A graph of stiffness resolution versus initial thickness for different acoustic pressure values is given is Supplementary Figure S22. It is seen that the accuracy increases as we increase the applied acoustic pressure. In practice, however, the maximum pressure value that can be applied is limited with the transducer and cell's viability as we discussed in the main text.

# *S.2. Supplementary Figures*

**Supplementary Figure S1.** Flowchart of the stiffness map reconstruction

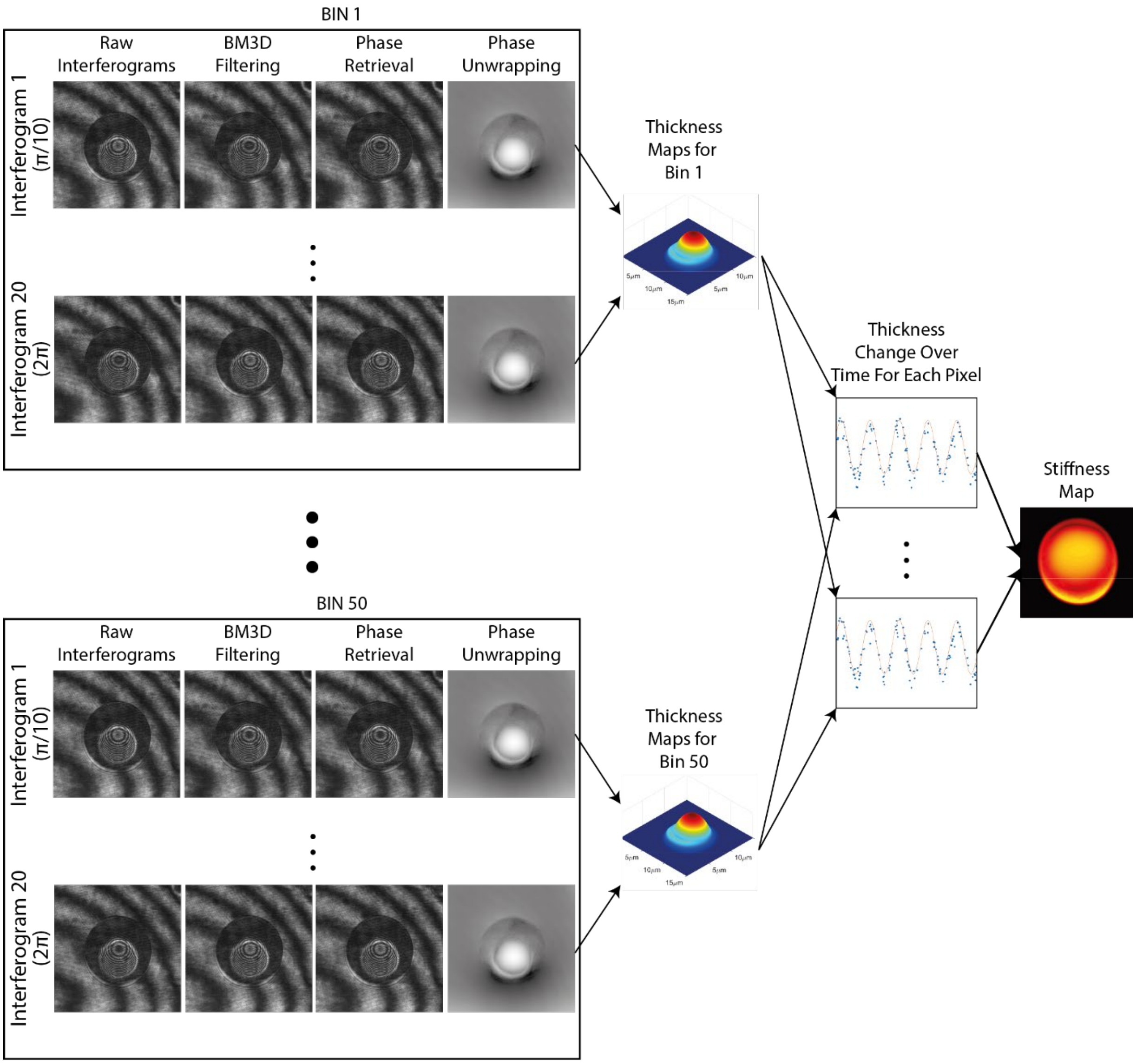

**Supplementary Figure S2.** Interferograms obtained at 0, π/2, π, 3π/2, 2π phase shifts for **a**, a polyacrylamide (PAA) microbead, **b**, an agarose microbead, **c**, an epithelial HCT-116 cell, and **d**, a TGF-β treated HCT-116 cell.

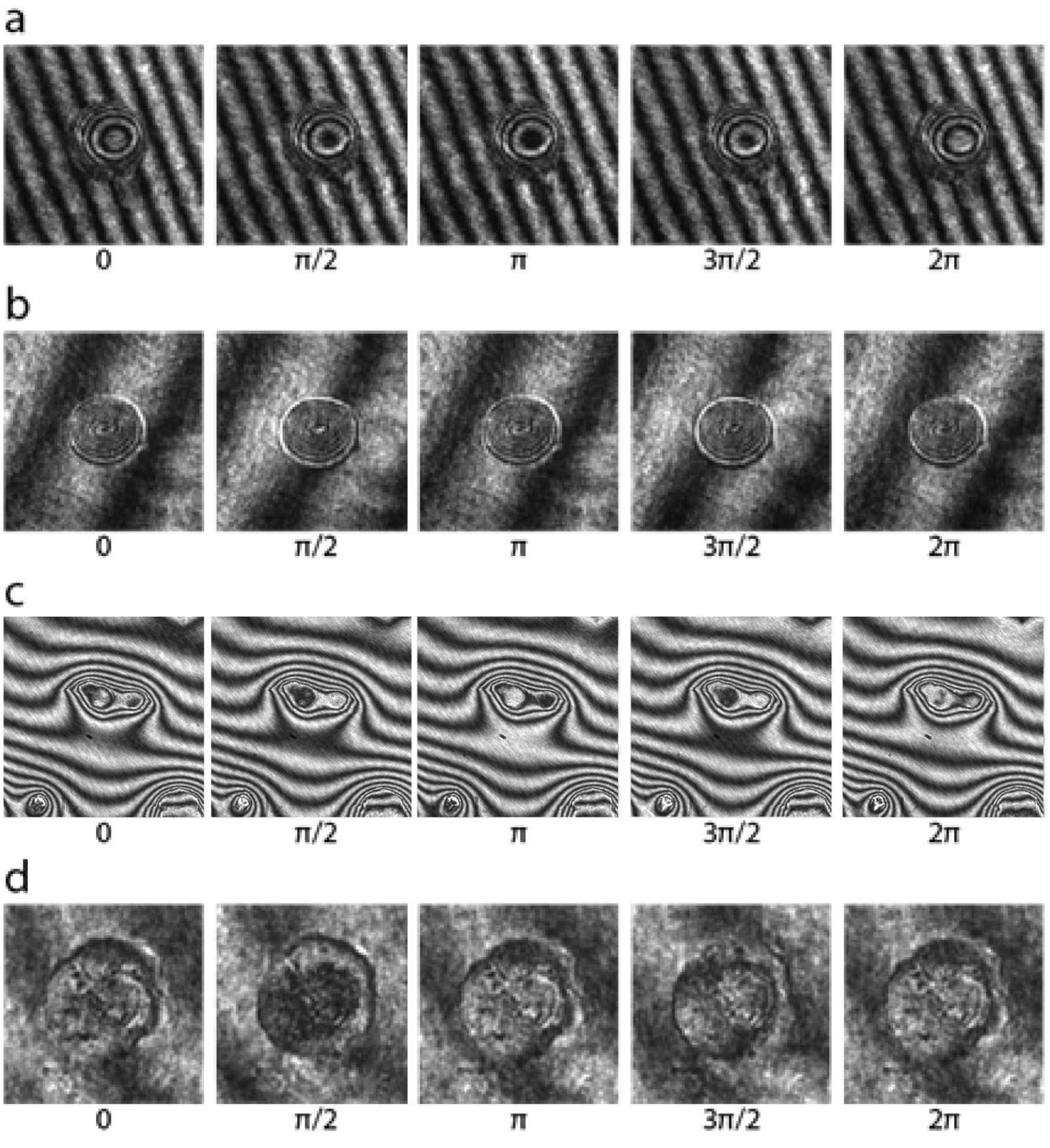

**Supplementary Figure S3.** Stiffness measurement of a polystyrene (PS) microbead obtained using the acousto-holographic method. **a** Amplitude image of the microbead. **b** Thickness map of the microbead obtained through the reconstruction of the captured interferograms. **c** Reconstructed displacement of a microbead obtained by measuring the change in thickness of a certain point on its surface relative to the initial thickness. **d** Stiffness distribution of the microbead obtained from displacement measurements of various points on the microbead using the Hertzian model.

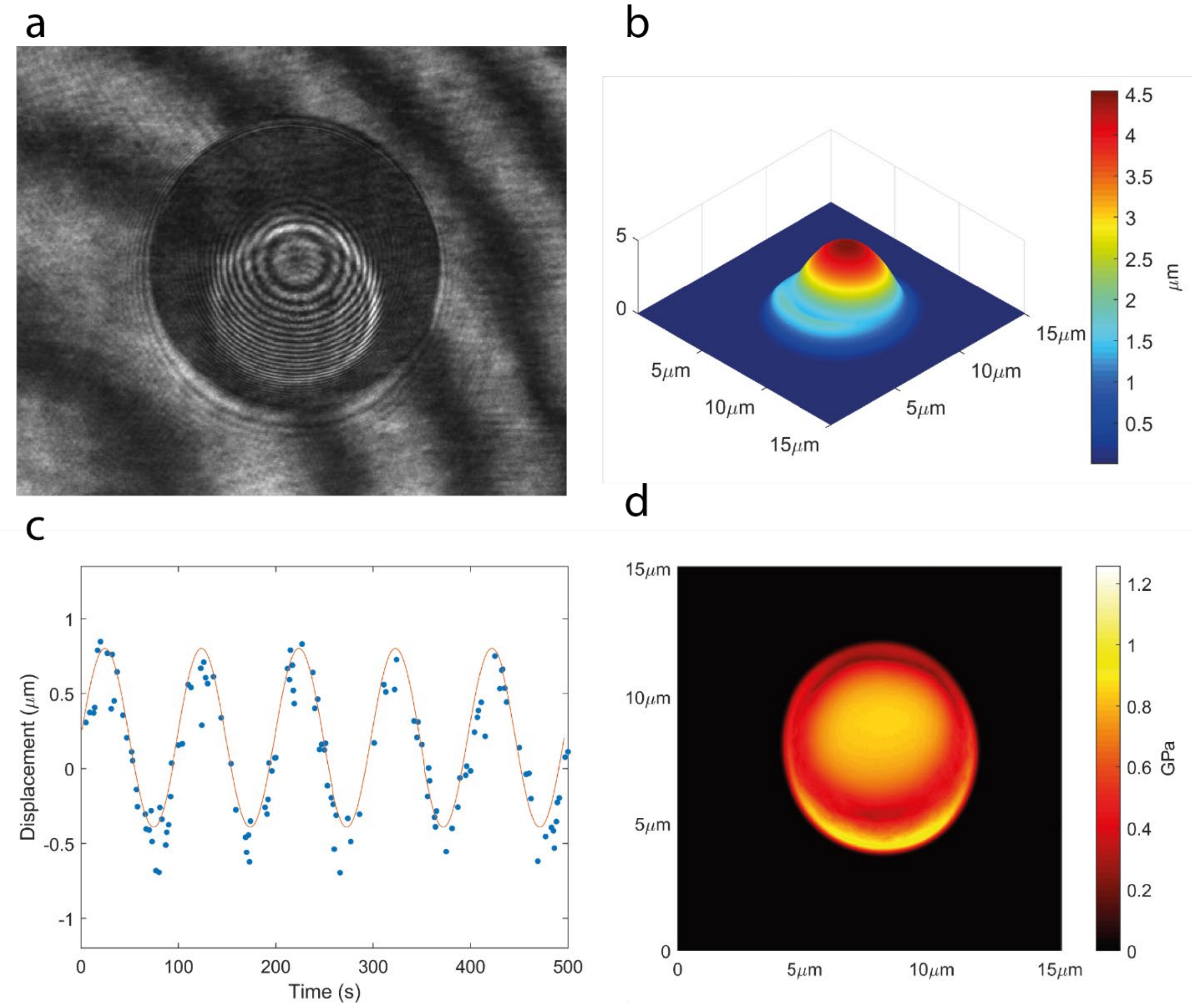

**Supplementary Figure S4.** Stiffness distributions of three different HCT116 cells measured using AFM indentation. Calculated average stiffness of **a** 1.10 kPa, **b** 1.26 kPa, and **c** 1.18 kPa are in good agreement with the values obtained using the acousto-holographic method.

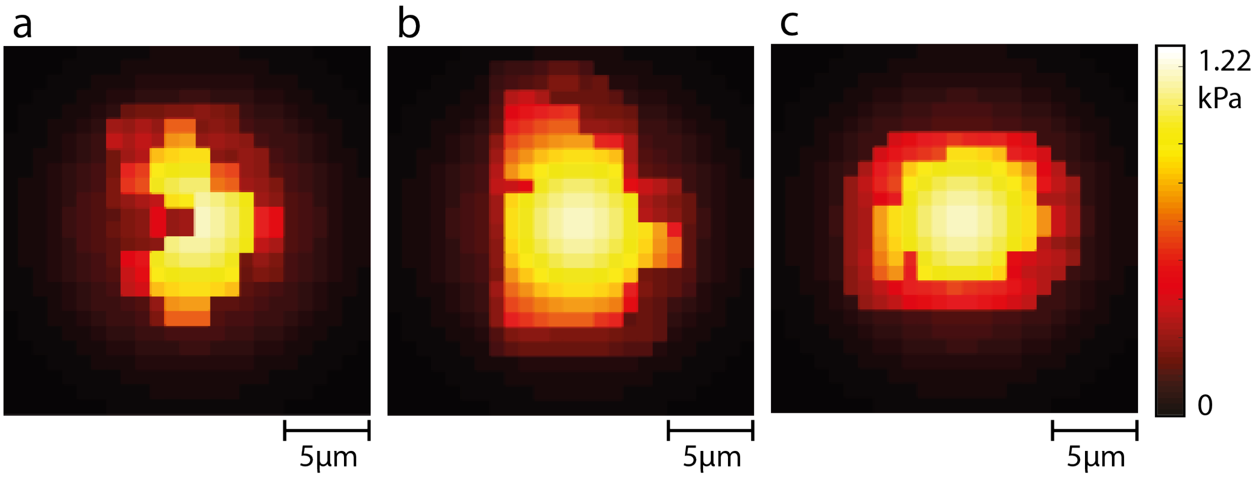


**Supplementary Figure S5.** Inverted microscope images of **a** a polyacrylamide (PAA) and **b** an agarose microbead.

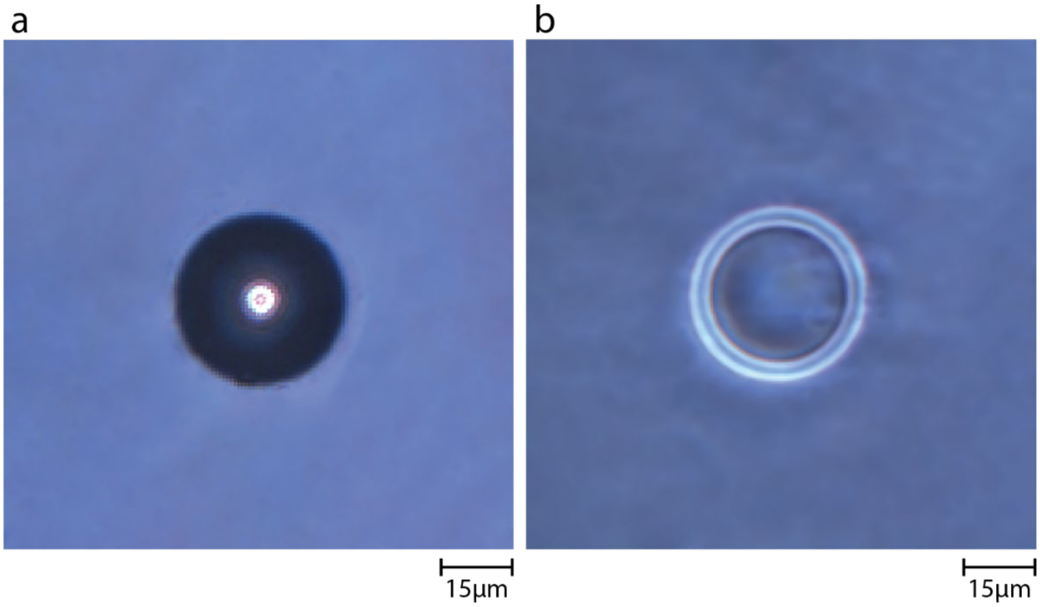

**Supplementary Figure S6.** Stiffness maps obtained from three different **a** PAA, **b** agarose, and **c** PS microbeads within deionized water (left), DMEM-F12 (middle), and glycerol (right). Shape distortion is clearly seen for the PS microbead measured in deionized water due to the microbead's high refractive index. Such distortions are suppressed in the glycerol which has a high refractive index medium.

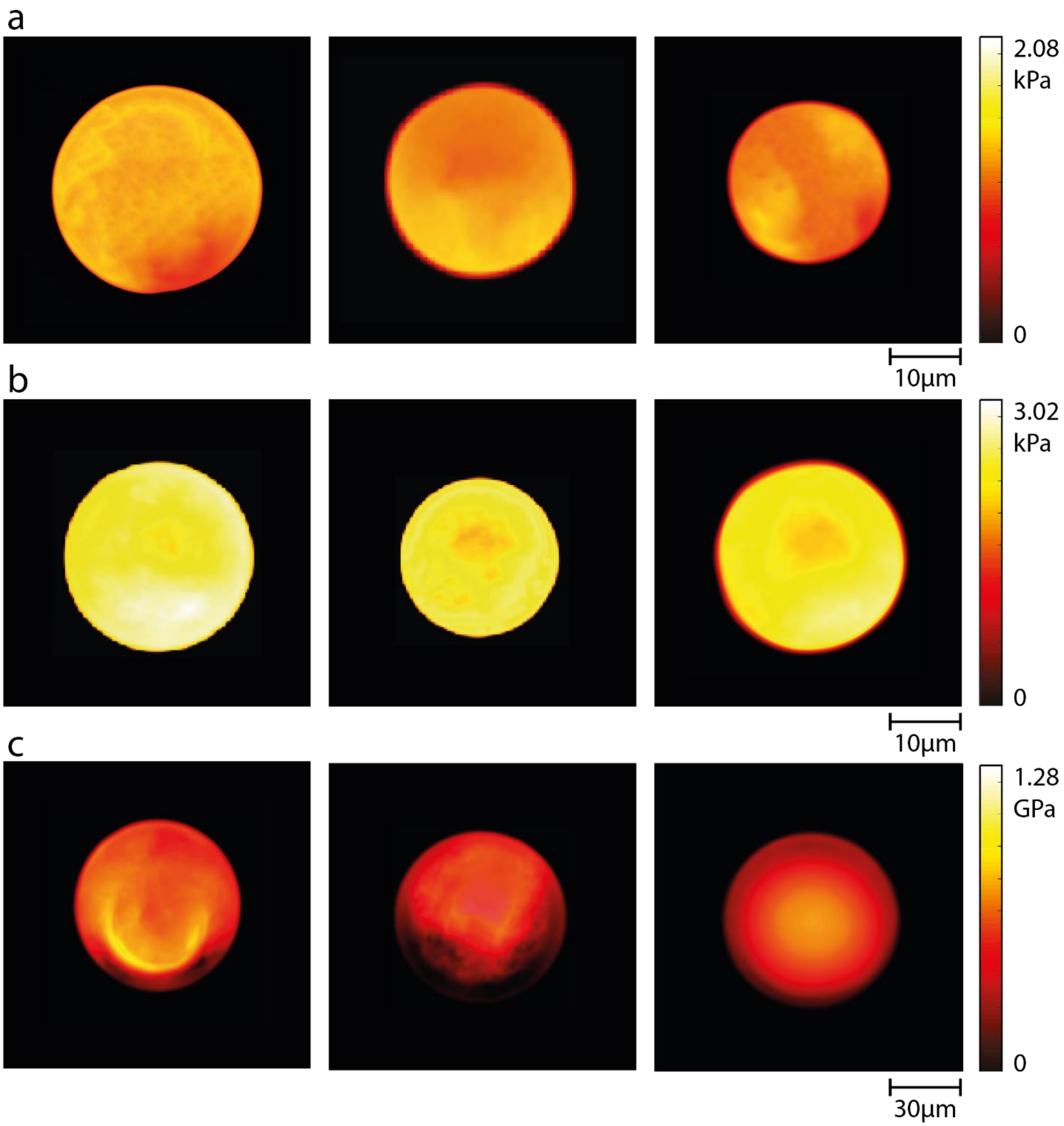

**Supplementary Figure S7.** Thickness maps for **a** PAA microbeads, **b** agarose microbeads, and **c** polystyrene microbeads within deionized water (left), DMEM-F12 (middle), and glycerol (right). A similar pattern where high refractive index disparity causes measurement distortions can be observed here for polystyrene microbeads.

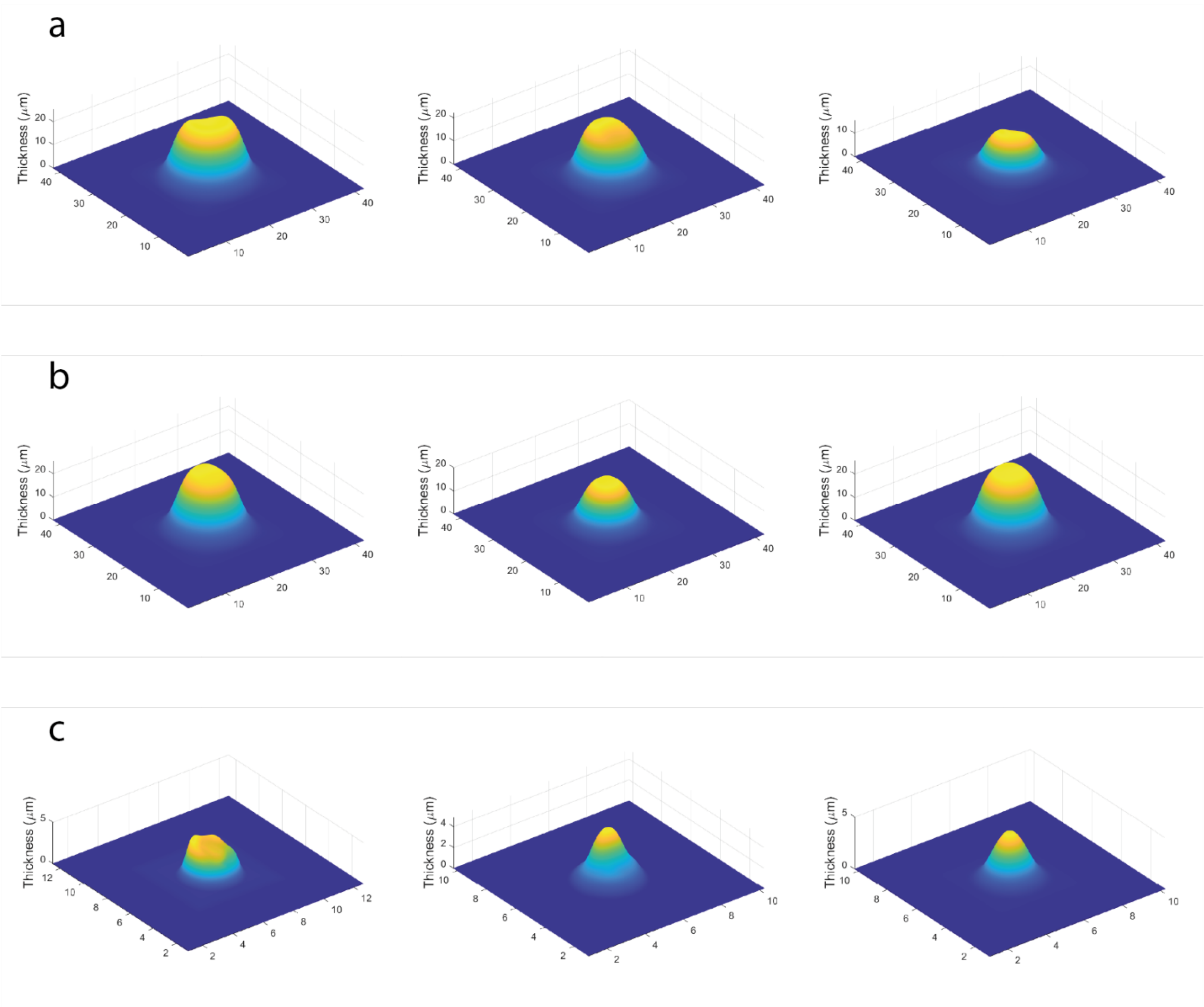

**Supplementary Figure S8.** 2D stiffness map obtained for a PAA microbead using AFM measurements and vertical and horizontal cross-sections (solid lines) of the stiffness through the centre of the image.

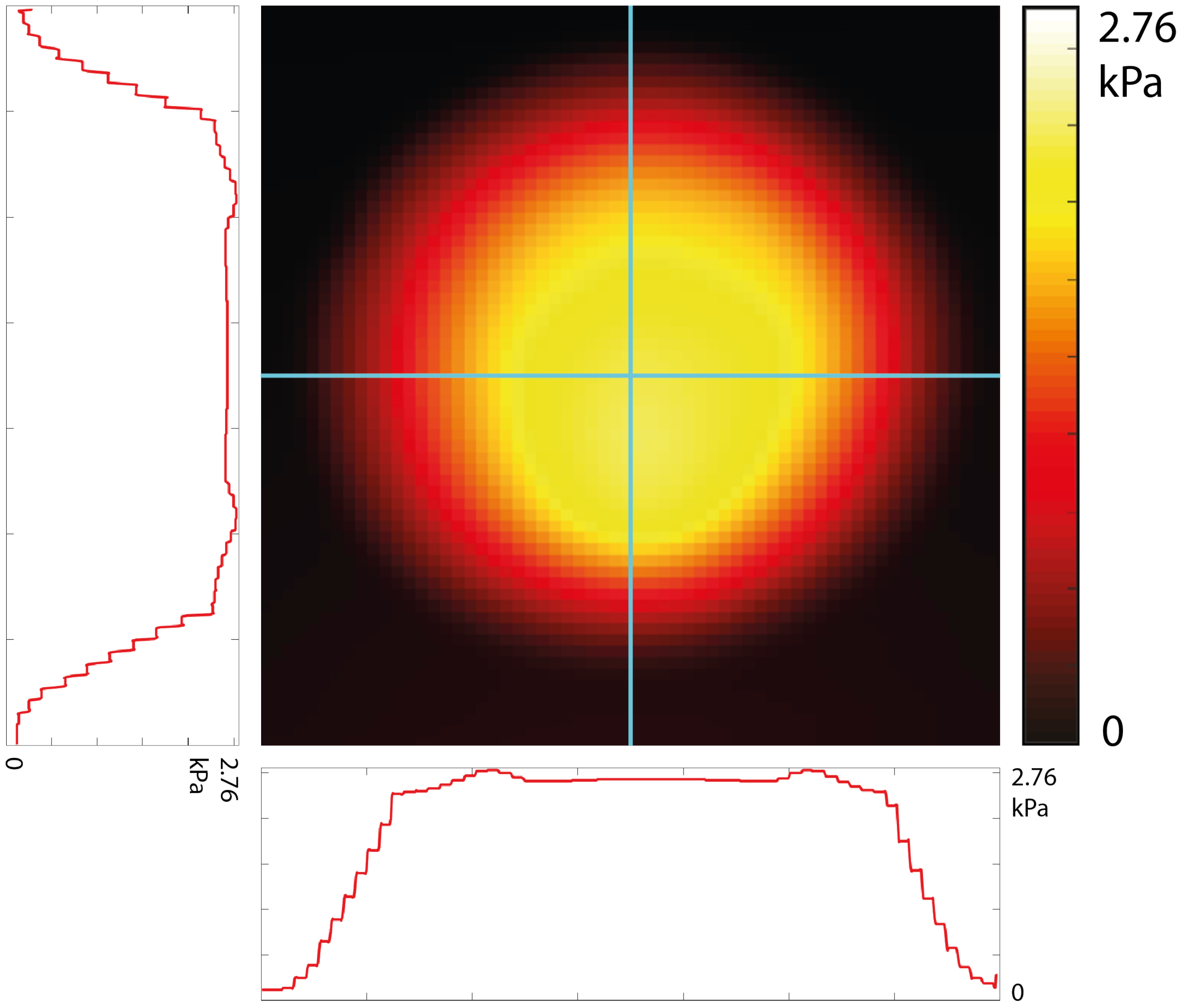

**Supplementary Figure S9.** 2D stiffness map obtained for a PAA microbead using the acousto-holographic measurement system and vertical and horizontal cross-sections (solid lines) of the stiffness through the centre of the image.

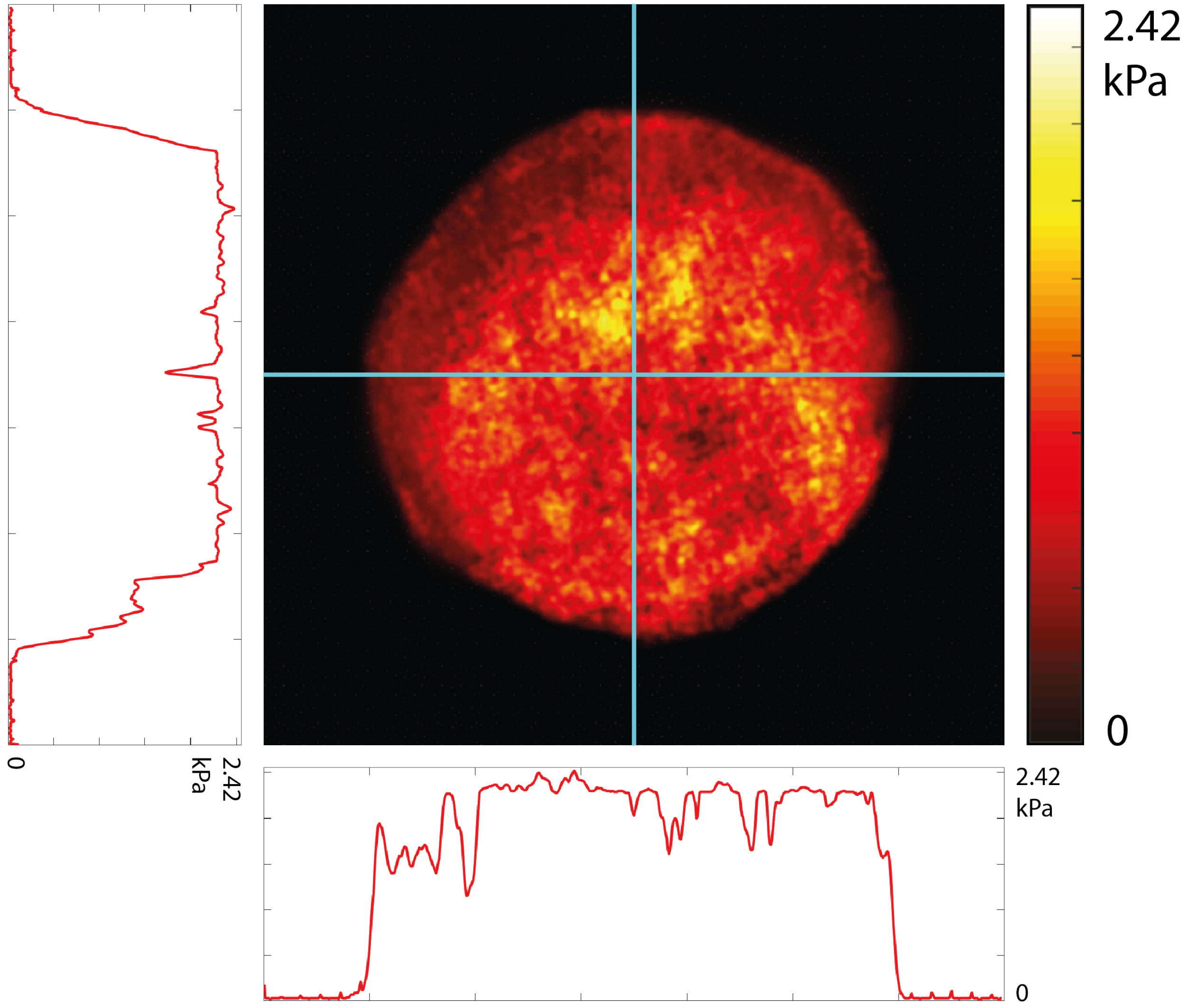

**Supplementary Figure S10.** 2D stiffness map obtained for an agarose microbead using AFM measurements and vertical and horizontal cross-sections (solid lines) of the stiffness through the centre of the image.

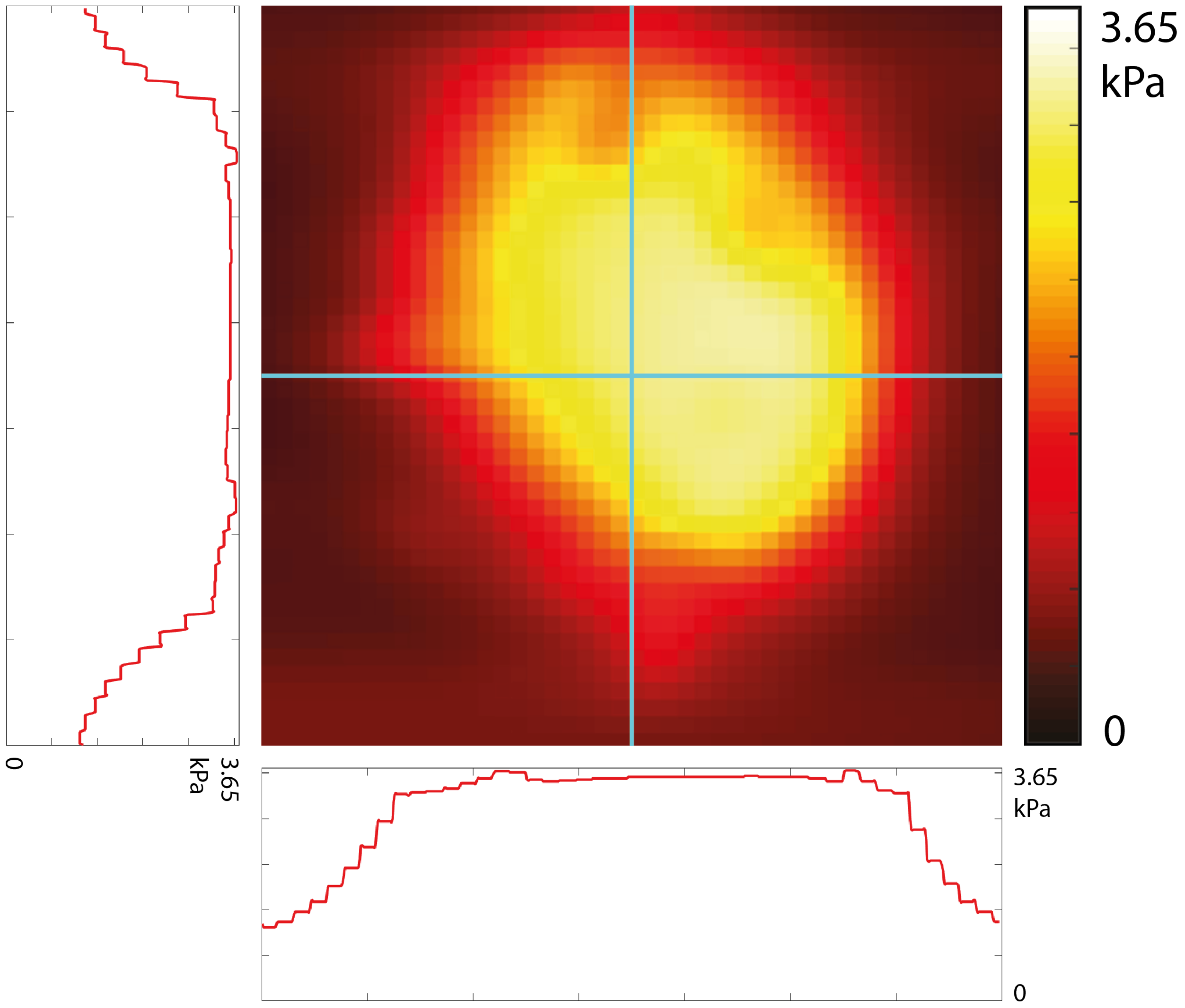

**Supplementary Figure S11.** 2D stiffness map obtained for an agarose microbead using the acousto-holographic measurement system and vertical and horizontal cross-sections (solid lines) of the stiffness through the centre of the image.

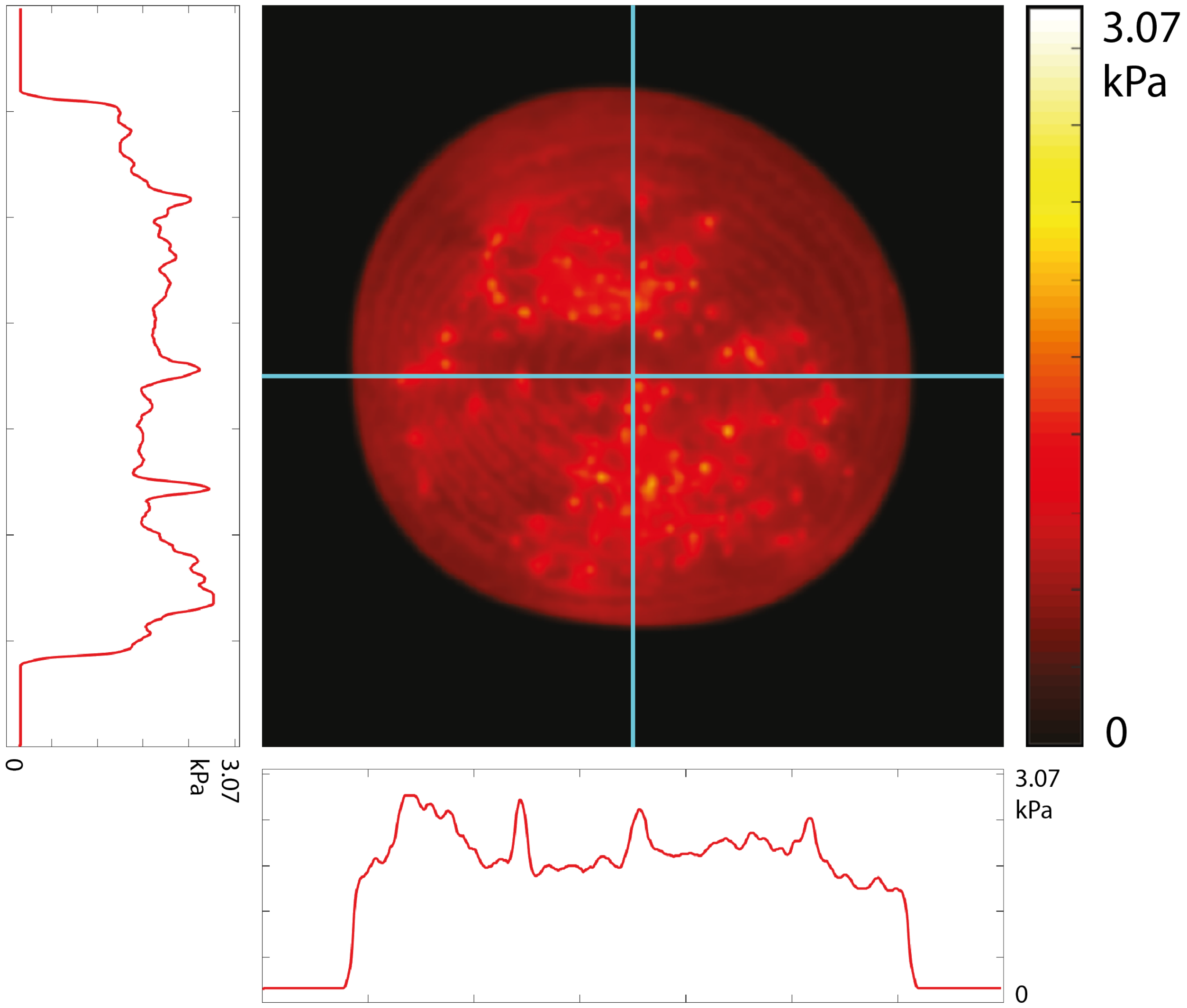

**Supplementary Figure S12.** A schematic describing the finite element analysis (FEA) conducted using COMSOL Multiphysics. **a** The geometric structure, **b** the phase shifted interferograms obtained in COMSOL, **c** thickness maps calculated in MATLAB, **d** stiffness map calculated in MATLAB.

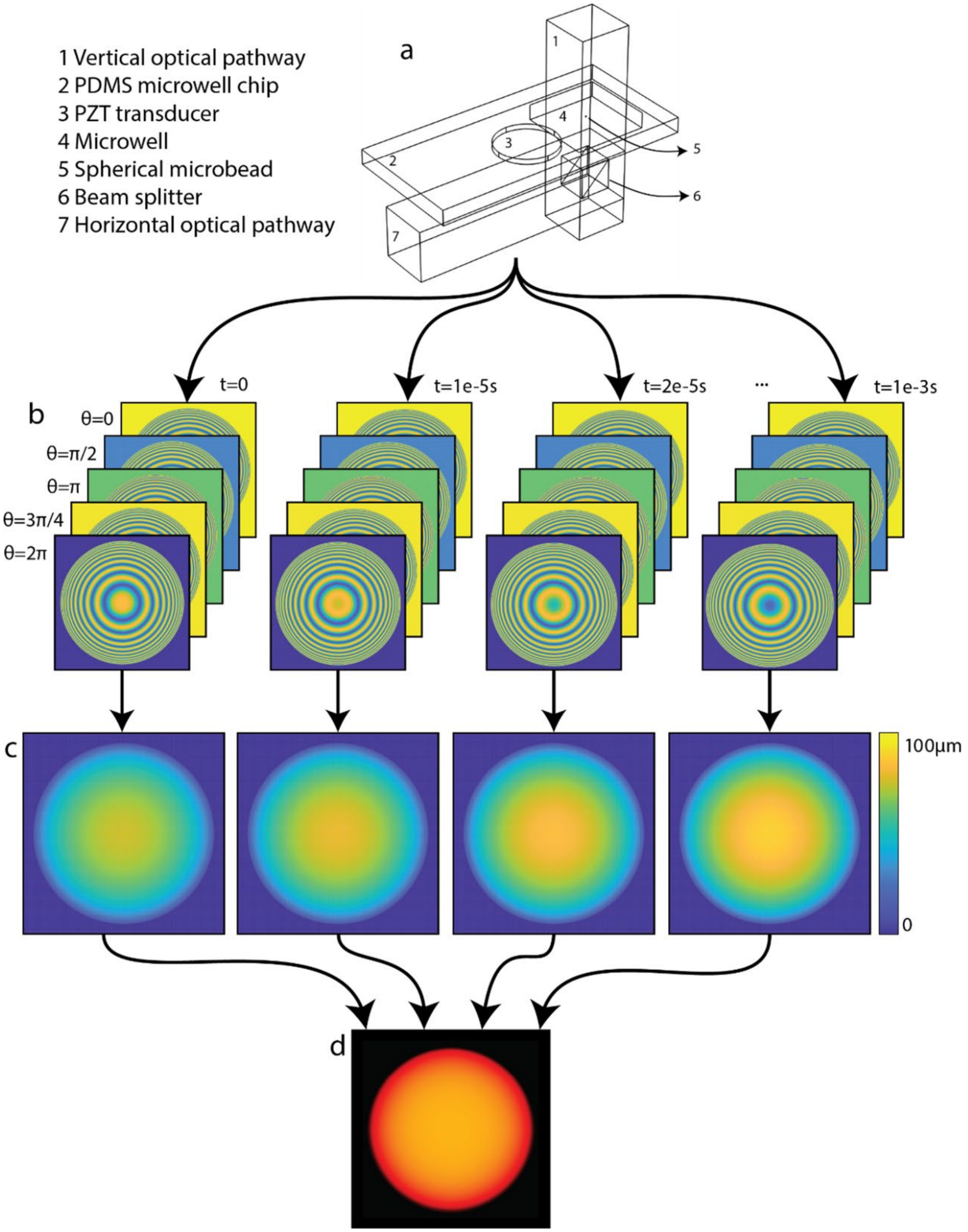

**Supplementary Figure S13.** Comparison of stiffness maps obtained from FEA in COMSOL (right) and from acousto-holographic measurement system (left) for **a** PAA, **b** agarose, and **c** PS microbeads.

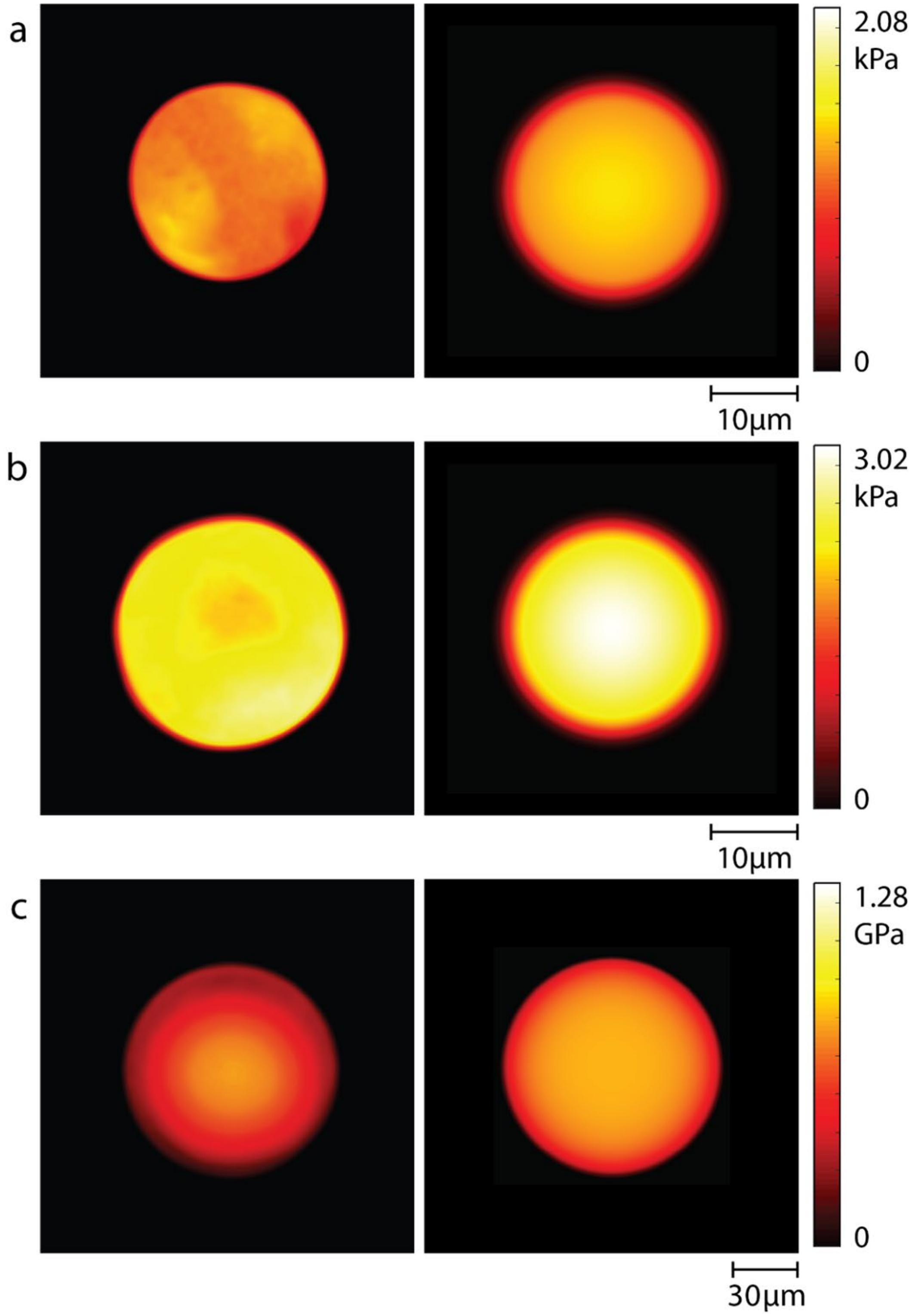

**Supplementary Figure S14.** Histograms showing the stiffness distributions obtained from the reconstructed 2D stiffness maps obtained for PAA microbeads taken from the same batch and measured using **a** acousto-holographic system and **b** AFM. **c** presents the result obtained from COMSOL simulations for a PAA microbead whose size is the average size of the microbeads in the batch used in the experiments.

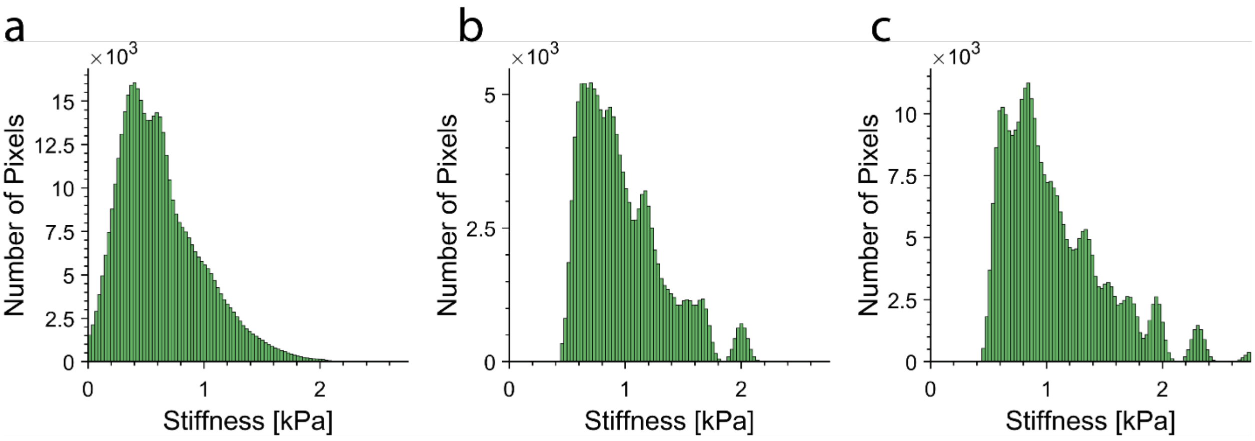


**Supplementary Figure S15.** Histograms showing the stiffness distributions obtained from the reconstructed 2D stiffness maps obtained for agarose microbeads taken from the same batch and measured using **a** acousto-holographic system and **b** AFM. **c** presents the result obtained from COMSOL simulations for an agarose microbead whose size is the average size of the microbeads in the batch used in the experiments.

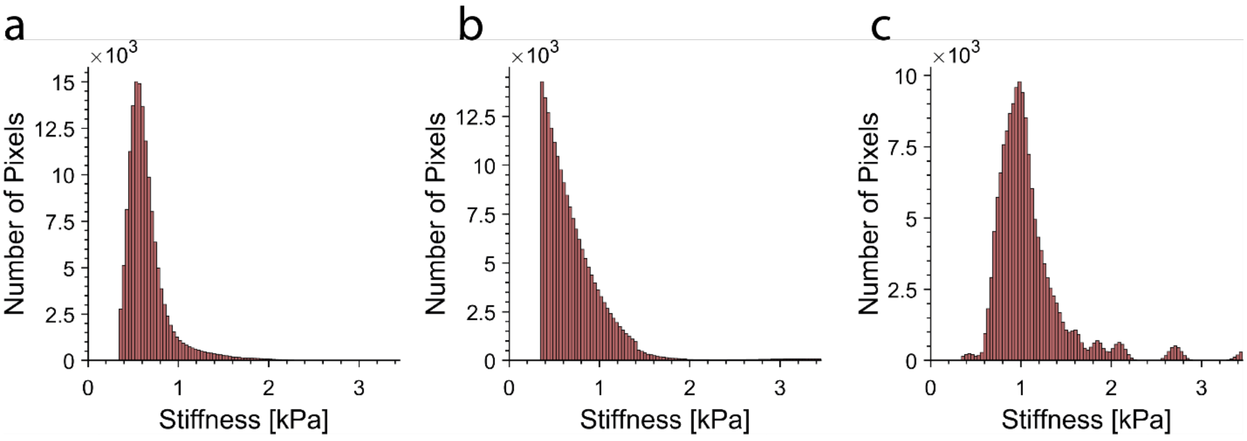

**Supplementary Figure S16.** Histograms showing the stiffness distributions from the reconstructed 2D stiffness maps obtained for PS microbeads measured using **a** acousto-holographic system and **b** COMSOL simulations with a PS microbead whose size is the average size of the microbeads in the batch used in the experiments.

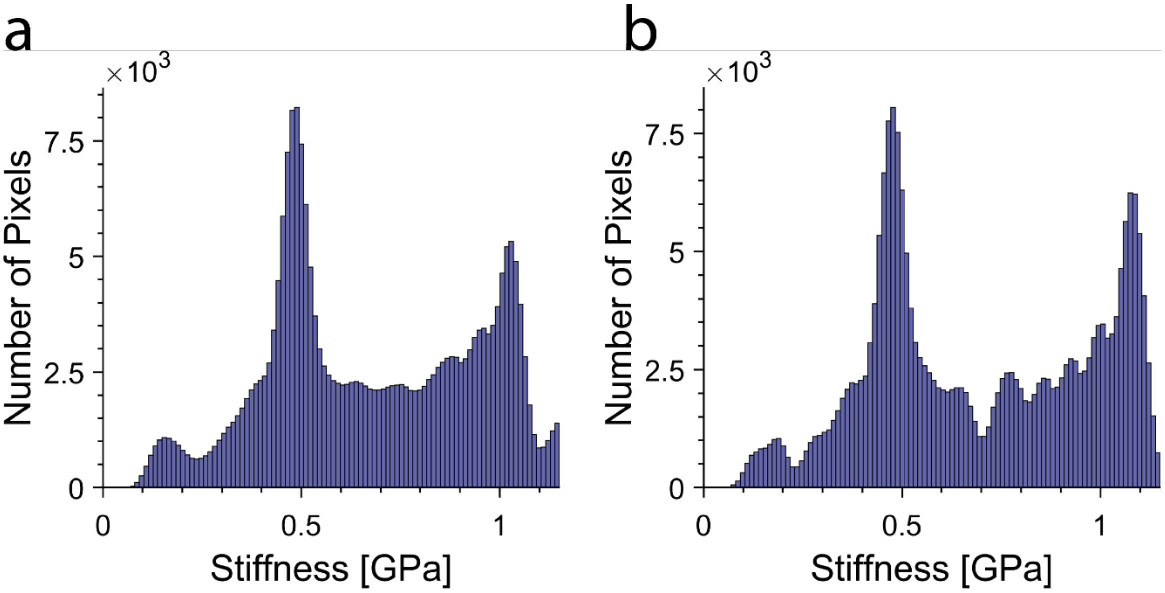


**Supplementary Figure S17.** Histograms showing the stiffness distributions from the reconstructed 2D stiffness maps obtained for **a** epithelial HCT116 cells using acousto-holographic system, **b** epithelial HCT116 cells using AFM measurements, and **c** CTC-mimicking HCT116 cells using acousto-holographic system.

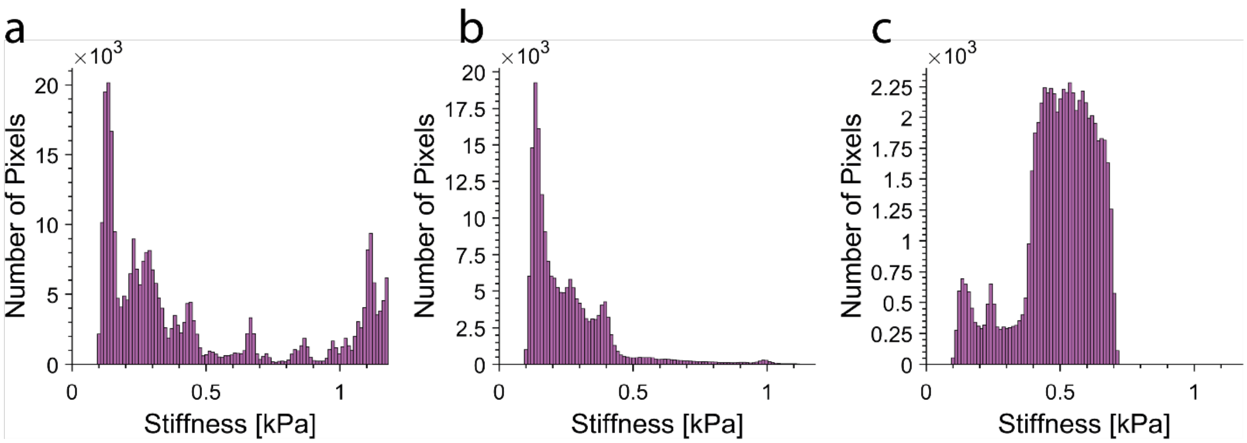

**Supplementary Figure S18.** Boxplots showing the distribution of $R^2$ values calculated by comparing stiffness histograms obtained from 2D stiffness maps. **a-c** Comparison of the results obtained from the acousto-holographic and AFM measurements of **a** PAA microbeads (Fig. S14), **b** agarose microbeads (Fig. S15), and **c** epithelial HCT116 cells (Fig. S17c). **d** Comparison of the results obtained from the acousto-holographic measurements of epithelial HCT116 cells (Fig. S17a) and CTC-mimicking cells (Figure S17c). Measurements with AFM and acousto-holographic system shows a very good similarity. Low similarity (i.e., high dissimilarity) between the results for epithelial HCT116 and CTC-mimicking cells obtained with acousto-holographic system implies the large stiffness difference between these two cells, as expected. The red line denotes the median value, the rectangular box denotes the range between 25$^{th}$ percentile and 75$^{th}$ percentile, and the whiskers denote the range between 0$^{th}$ and 100$^{th}$ percentile. For **a** and **b**, 50 comparisons were made (1 obtained from AFM against 50 obtained from acousto-holographic system over three independent experiments). For **c**, 1225 comparisons were made (35 from AFM and 35 from acousto-holographic system both over three independent experiments). For **d**, 875 comparisons were made (35 epithelial and 25 CTC-mimicking HCT116 cells, both measured using acousto-holographic system over three independent experiments).

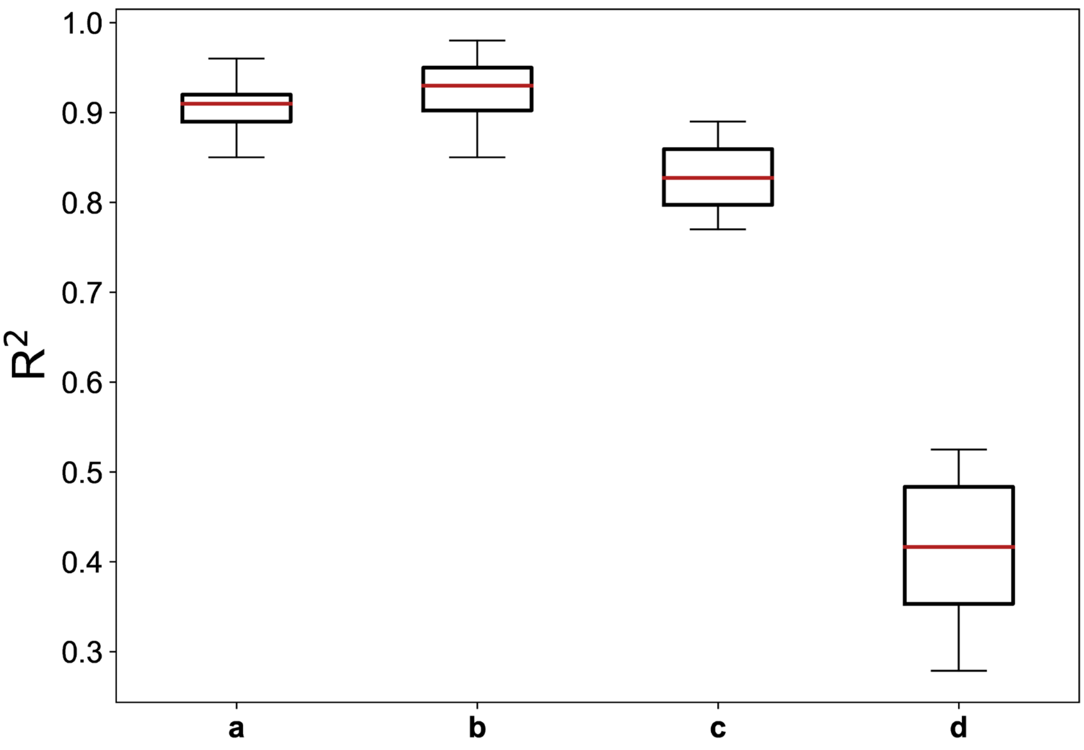

**Supplementary Figure S19.** Reconstructed stiffness maps of PS microbeads **a** 15$\mu$m diameter and **b** 100$\mu$m diameter and the calculation of the two-point resolution from their spatial dimensions. Both microbeads yield similar resolution values that are reasonably close to the theoretical resolution.

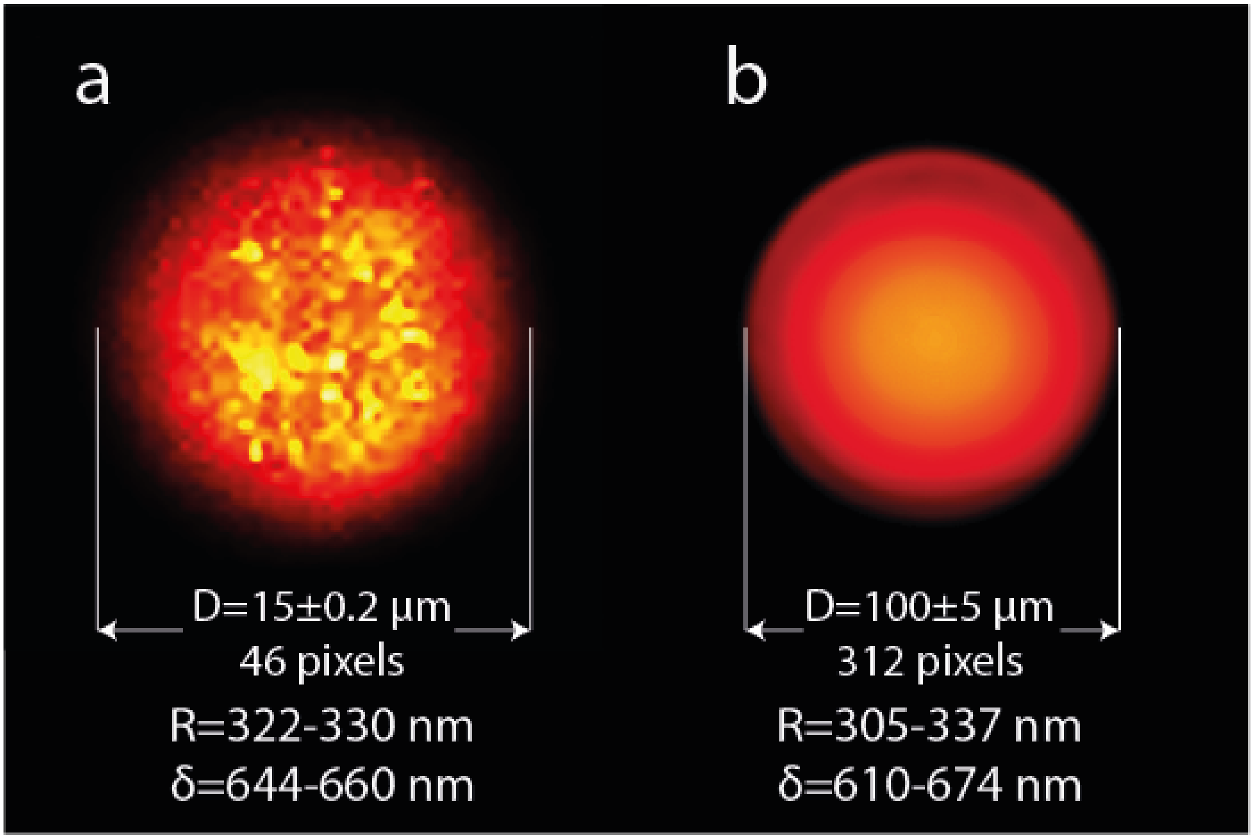

**Supplementary Figure S20.** Distribution of thickness, diameter and stiffness values calculated from an ensemble of n=50 PS microbeads of 15 $\mu$m diameter over three independent experiments. The red line denotes the median value, the rectangular box denotes the range between 25$^{th}$ percentile and 75$^{th}$ percentile, and the whiskers denote the range between 0$^{th}$ and 100$^{th}$ percentile.

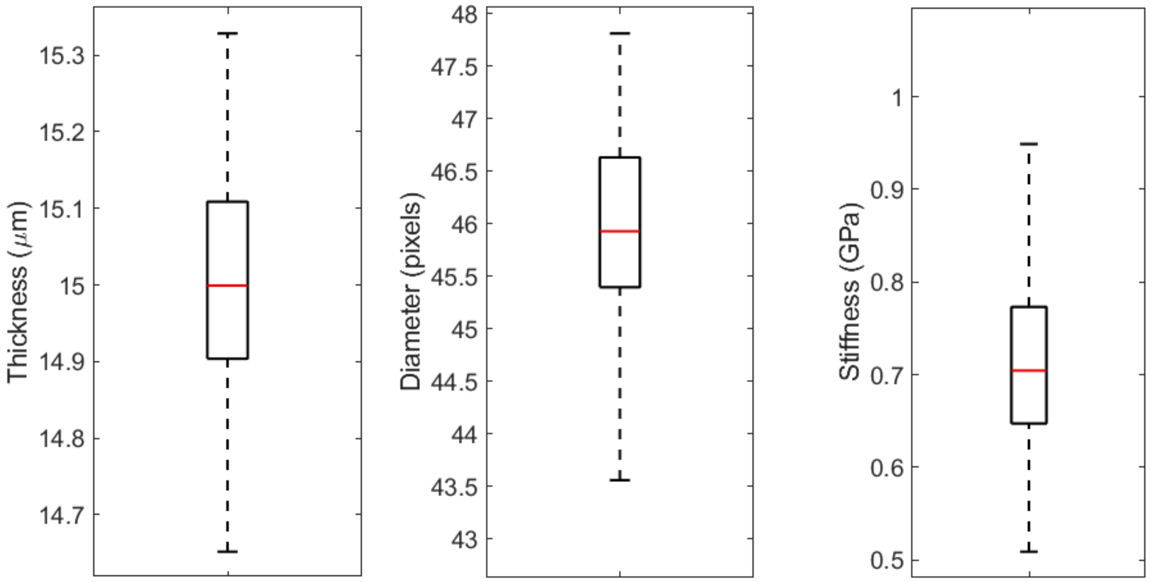

**Supplementary Figure S21.** 2D stiffness map obtained for a PS microbead of 15 $\mu$m diameter using the acousto-holographic measurement system and vertical and horizontal cross-sections (solid lines) of the stiffness through the centre of the image.

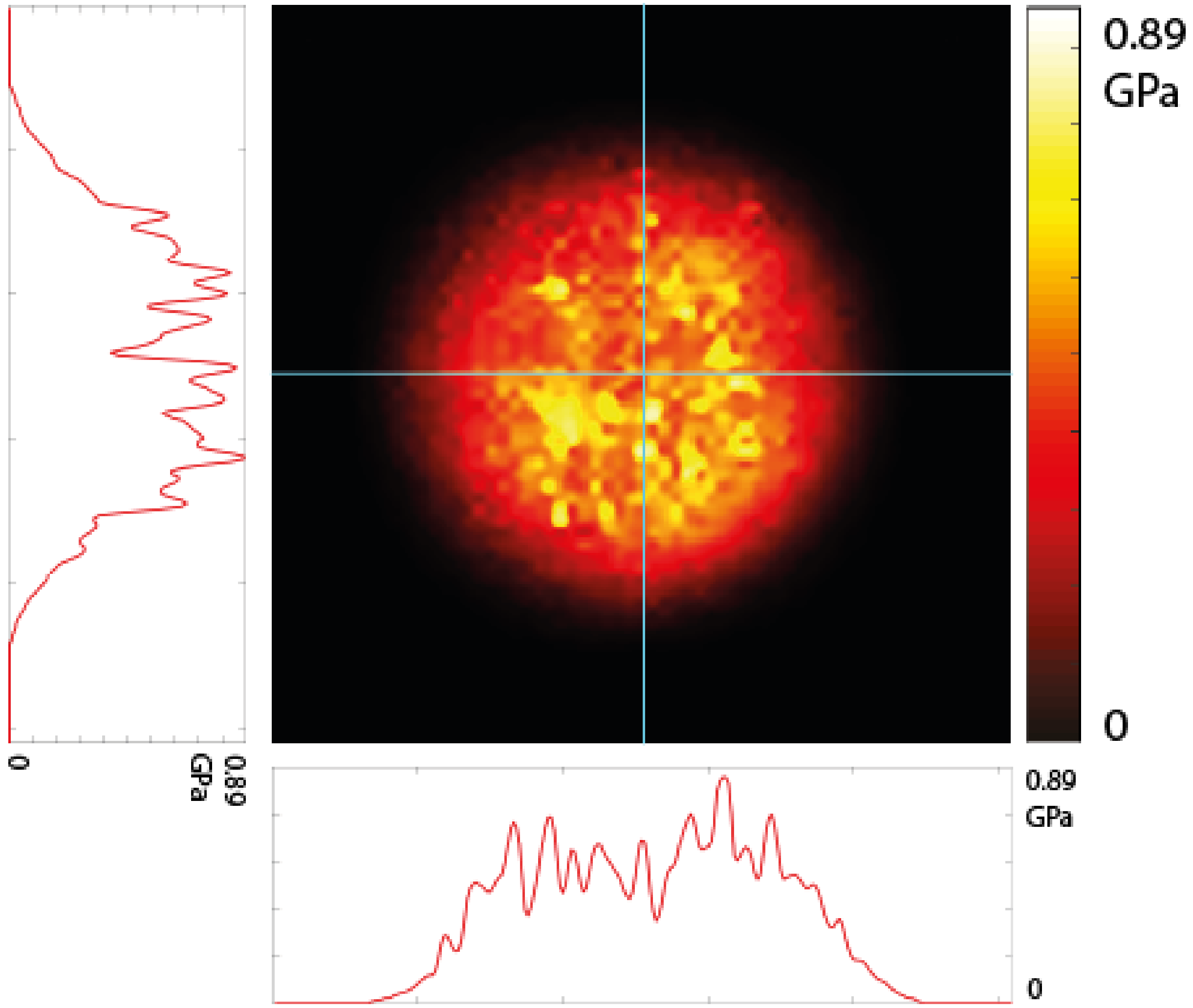

**Supplementary Figure S22.** Change in resolution against change in initial thickness for different acoustic pressure levels.

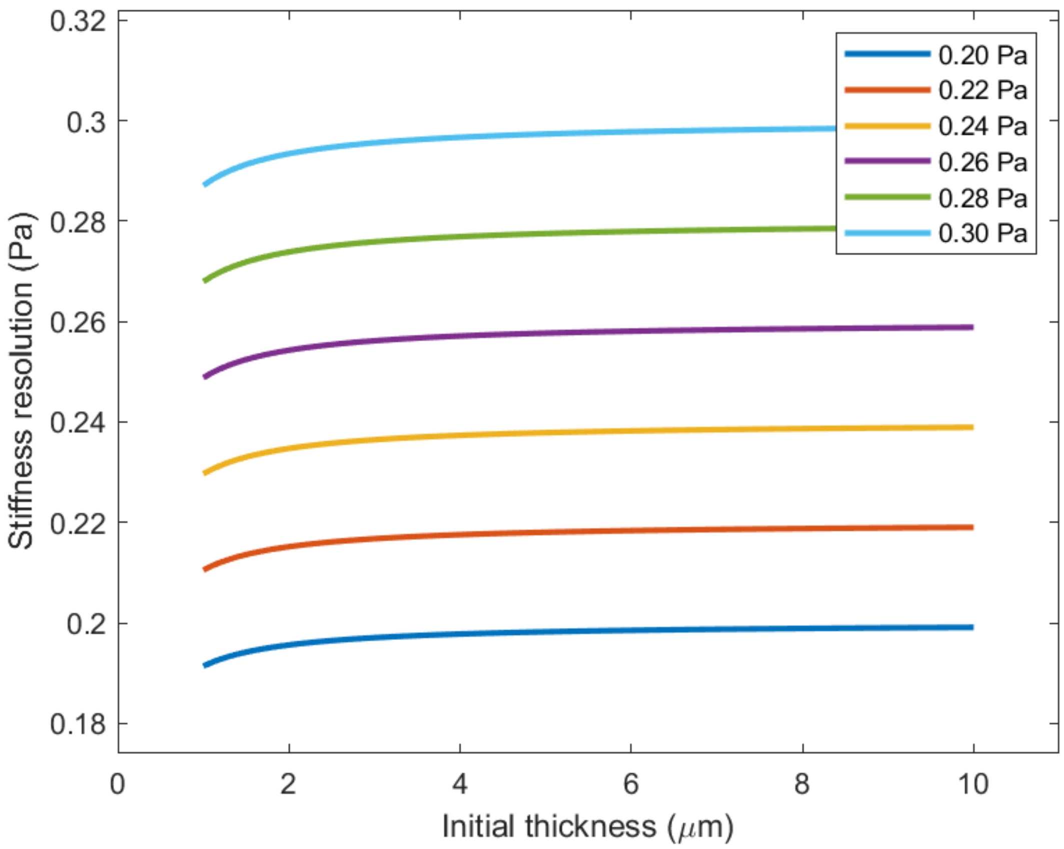

**Supplementary Table 1.** Average stiffness over the whole microbead and its variation obtained from 10 consecutive measurements performed on the same PAA, agarose, and PS microbeads in deionized (DI) water, DMEM-F12, and glycerol. Note that for each medium we used a different microbead from the batch.

| | **PAA bead in DI water** | **PAA bead in DMEM-F12** | **PAA bead in glycerol** | **Agarose bead in DI water** | **Agarose bead in DMEM-F12** | **Agarose bead in glycerol** | **PS bead in DI water** | **PS bead in DMEM-F12** | **PS bead in glycerol** |
|---|---|---|---|---|---|---|---|---|---|
| **Exp 1** | 1.85 kPa | 1.94 kPa | 2.13 kPa | 2.18 kPa | 2.46 kPa | 2.30 kPa | 0.94 GPa | 0.91 GPa | 1.14 GPa |
| **Exp 2** | 1.73 kPa | 2.04 kPa | 2.06 kPa | 2.53 kPa | 2.41 kPa | 2.67 kPa | 1.10 GPa | 1.09 GPa | 1.08 GPa |
| **Exp 3** | 1.95 kPa | 2.05 kPa | 1.89 kPa | 2.25 kPa | 2.44 kPa | 2.45 kPa | 1.01 GPa | 1.12 GPa | 1.08 GPa |
| **Exp 4** | 1.89 kPa | 2.04 kPa | 2.08 kPa | 2.45 kPa | 2.55 kPa | 2.34 kPa | 1.10 GPa | 0.91 GPa | 1.12 GPa |
| **Exp 5** | 1.89 kPa | 1.89 kPa | 2.04 kPa | 2.50 kPa | 2.45 kPa | 2.39 kPa | 0.90 GPa | 0.94 GPa | 1.07 GPa |
| **Exp 6** | 1.67 kPa | 2.09 kPa | 2.18 kPa | 2.54 kPa | 2.34 kPa | 2.34 kPa | 0.92 GPa | 0.96 GPa | 1.13 GPa |
| **Exp 7** | 2.07 kPa | 1.91 kPa | 1.94 kPa | 2.38 kPa | 2.39 kPa | 2.30 kPa | 1.14 GPa | 0.95 GPa | 1.11 GPa |
| **Exp 8** | 2.15 kPa | 2.18 kPa | 1.69 kPa | 2.61 kPa | 2.50 kPa | 2.27 kPa | 0.94 GPa | 0.95 GPa | 1.08 GPa |
| **Exp 9** | 2.13 kPa | 1.79 kPa | 2.01 kPa | 2.44 kPa | 2.23 kPa | 2.22 kPa | 1.03 GPa | 0.91 GPa | 1.09 GPa |
| **Exp 10** | 2.16 kPa | 2.12 kPa | 1.78 kPa | 2.49 kPa | 2.46 kPa | 2.52 kPa | 0.99 GPa | 0.94 GPa | 1.12 GPa |
| **Avg.** | 1.95 kPa | 2.01 kPa | 1.98 kPa | 2.44 kPa | 2.42 kPa | 2.38 kPa | 1.01 GPa | 0.97 GPa | 1.10 GPa |
| **Std. Dev.** | 0.17 kPa | 0.12 kPa | 0.16 kPa | 0.13 kPa | 0.09 kPa | 0.13 kPa | 0.08 GPa | 0.07 GPa | 0.02 GPa |

**Supplementary Table 2.** Coefficient of determination $R^2$ values calculated by comparing the histograms of stiffness distributions from simulations and acousto-holographic measurements.

| | PS Beads | PAA Beads | Agarose Beads | | PS Beads | PAA Beads | Agarose Beads |
|---|---|---|---|---|---|---|---|
| **Sample 1** | 0.90 | 0.91 | 0.90 | **Sample 26** | 0.91 | 0.90 | 0.89 |
| **Sample 2** | 0.91 | 0.89 | 0.87 | **Sample 27** | 0.91 | 0.89 | 0.87 |
| **Sample 3** | 0.90 | 0.87 | 0.88 | **Sample 28** | 0.92 | 0.88 | 0.88 |
| **Sample 4** | 0.90 | 0.87 | 0.89 | **Sample 29** | 0.90 | 0.89 | 0.88 |
| **Sample 5** | 0.91 | 0.90 | 0.89 | **Sample 30** | 0.91 | 0.90 | 0.90 |
| **Sample 6** | 0.91 | 0.89 | 0.86 | **Sample 31** | 0.91 | 0.89 | 0.88 |
| **Sample 7** | 0.90 | 0.90 | 0.86 | **Sample 32** | 0.91 | 0.88 | 0.88 |
| **Sample 8** | 0.90 | 0.88 | 0.86 | **Sample 33** | 0.91 | 0.91 | 0.89 |
| **Sample 9** | 0.92 | 0.88 | 0.88 | **Sample 34** | 0.92 | 0.88 | 0.89 |
| **Sample 10** | 0.91 | 0.90 | 0.89 | **Sample 35** | 0.90 | 0.91 | 0.88 |
| **Sample 11** | 0.92 | 0.90 | 0.90 | **Sample 36** | 0.91 | 0.89 | 0.89 |
| **Sample 12** | 0.91 | 0.89 | 0.90 | **Sample 37** | 0.91 | 0.88 | 0.88 |
| **Sample 13** | 0.91 | 0.87 | 0.88 | **Sample 38** | 0.91 | 0.87 | 0.87 |
| **Sample 14** | 0.91 | 0.90 | 0.90 | **Sample 39** | 0.91 | 0.90 | 0.89 |
| **Sample 15** | 0.91 | 0.88 | 0.87 | **Sample 40** | 0.91 | 0.88 | 0.87 |
| **Sample 16** | 0.92 | 0.88 | 0.86 | **Sample 41** | 0.92 | 0.90 | 0.89 |
| **Sample 17** | 0.90 | 0.87 | 0.90 | **Sample 42** | 0.90 | 0.89 | 0.89 |
| **Sample 18** | 0.92 | 0.88 | 0.90 | **Sample 43** | 0.92 | 0.91 | 0.87 |
| **Sample 19** | 0.91 | 0.87 | 0.88 | **Sample 44** | 0.90 | 0.88 | 0.90 |
| **Sample 20** | 0.91 | 0.90 | 0.86 | **Sample 45** | 0.91 | 0.88 | 0.87 |
| **Sample 21** | 0.91 | 0.88 | 0.90 | **Sample 46** | 0.90 | 0.90 | 0.88 |
| **Sample 22** | 0.92 | 0.91 | 0.90 | **Sample 47** | 0.91 | 0.89 | 0.88 |
| **Sample 23** | 0.91 | 0.89 | 0.86 | **Sample 48** | 0.92 | 0.88 | 0.88 |
| **Sample 24** | 0.92 | 0.89 | 0.89 | **Sample 49** | 0.91 | 0.88 | 0.89 |
| **Sample 25** | 0.92 | 0.88 | 0.88 | **Sample 50** | 0.90 | 0.89 | 0.90 |
| | | | | Average | 0.91 | 0.89 | 0.88 |
| | | | | Std. Dev. | 0.02 | 004 | 0.05 |

**Supplementary Table 3.** Coefficient of determination $R^2$ values calculated by comparing the histograms of stiffness distributions obtained from acousto-holographic and AFM.

| | PAA Beads | Agarose Beads | | PAA Beads | Agarose Beads |
|---|---|---|---|---|---|
| **Sample 1** | 0.92 | 0.96 | **Sample 26** | 0.92 | 0.90 |
| **Sample 2** | 0.89 | 0.96 | **Sample 27** | 0.96 | 0.90 |
| **Sample 3** | 0.90 | 0.94 | **Sample 28** | 0.88 | 0.90 |
| **Sample 4** | 0.89 | 0.96 | **Sample 29** | 0.91 | 0.94 |
| **Sample 5** | 0.92 | 0.95 | **Sample 30** | 0.88 | 0.97 |
| **Sample 6** | 0.89 | 0.91 | **Sample 31** | 0.93 | 0.94 |
| **Sample 7** | 0.88 | 0.94 | **Sample 32** | 0.93 | 0.95 |
| **Sample 8** | 0.92 | 0.92 | **Sample 33** | 0.87 | 0.97 |
| **Sample 9** | 0.90 | 0.90 | **Sample 34** | 0.94 | 0.85 |
| **Sample 10** | 0.91 | 0.94 | **Sample 35** | 0.88 | 0.92 |
| **Sample 11** | 0.90 | 0.93 | **Sample 36** | 0.91 | 0.94 |
| **Sample 12** | 0.91 | 0.92 | **Sample 37** | 0.93 | 0.95 |
| **Sample 13** | 0.85 | 0.93 | **Sample 38** | 0.91 | 0.89 |
| **Sample 14** | 0.91 | 0.95 | **Sample 39** | 0.95 | 0.89 |
| **Sample 15** | 0.94 | 0.94 | **Sample 40** | 0.96 | 0.95 |
| **Sample 16** | 0.93 | 0.93 | **Sample 41** | 0.91 | 0.92 |
| **Sample 17** | 0.92 | 0.93 | **Sample 42** | 0.87 | 0.94 |
| **Sample 18** | 0.90 | 0.93 | **Sample 43** | 0.90 | 0.90 |
| **Sample 19** | 0.91 | 0.90 | **Sample 44** | 0.89 | 0.88 |
| **Sample 20** | 0.91 | 0.95 | **Sample 45** | 0.89 | 0.95 |
| **Sample 21** | 0.91 | 0.97 | **Sample 46** | 0.88 | 0.96 |
| **Sample 22** | 0.89 | 0.91 | **Sample 47** | 0.91 | 0.97 |
| **Sample 23** | 0.90 | 0.98 | **Sample 48** | 0.95 | 0.88 |
| **Sample 24** | 0.90 | 0.92 | **Sample 49** | 0.89 | 0.88 |
| **Sample 25** | 0.90 | 0.92 | **Sample 50** | 0.90 | 0.88 |
| | | | Average | 0.90 | 0.93 |
| | | | Std. Dev. | 0.03 | 0.03 |

## *Supplementary References*


1. Marston, P. L. & Apfel, R. E. Quadrupole resonance of drops driven by modulated acoustic radiation pressure—Experimental properties. *J. Acoust. Soc. Am.* **67**, 27–37 (1980).

2. Brillouin, L. *Les Tenseurs en Mécanique et en Élasticité*. (Dover Publications, New York, 1946).

3. Lee, C. P. & Wang, T. G. Acoustic radiation pressure. *J. Acoust. Soc. Am.* **94**, 1099–1109 (1993).

4. Wang, Z. & Han, B. Advanced iterative algorithm for phase extraction of randomly phase-shifted interferograms. *Opt. Lett.* **29**, 1671-1673 (2004).

5. Bazan, E., Dokládal, P. & Dokladalova, E. Quantitative Analysis of Similarity Measures of Distributions. in *British Machine Vision Conference 2019, BMVC 2019* (2019).

6. Kelly, D. P., Hennelly, B. M., Pandey, N., Naughton, T. J., & Rhodes, W. T. Resolution limits in practical digital holographic systems. *Opt. Eng.* **48**, 1–13 (2009)

7. Thomas, K. *Handbook of holographic interferometry: optical and digital methods*. (John Wiley & Sons, New York, 2006).

8. Abbe, E. Beiträge zur theorie des mikroskops und der mikroskopischen wahrnehmung. *Archiv für Mikroskopische Anatomie*, **9**, 413–418 (1873).

9. Goodman, J. W. *Introduction to Fourier Optics* (The McGraw-Hill Companies, New York, 1996).

10. Yamaguchi, I., Kato, J., Ohta, S., & Mizuno, J. Image formation in phase-shifting digital holography and applications to microscopy. *Appl. Opt.* **40**, 6177-6186 (2001).
