# Supplementary material for: Acousto-holographic reconstruction of whole-cell stiffness maps
Source: Nat Commun. 2022 Nov 29;13:7351. doi: 10.1038/s41467-022-35075-x (PMC9709086; doi:10.1038/s41467-022-35075-x)
Supplement: Supplementary file 2 — Reporting Summary [file 41467_2022_35075_MOESM2_ESM.pdf]

nature portfolio

Corresponding author(s): *Double-blind peer review submissions: write DBPR and your manuscript number here instead of author names.*

Last updated by author(s): *YYYY-MM-DD*

Reporting Summary

Nature Portfolio wishes to improve the reproducibility of the work that we publish. This form provides structure for consistency and transparency in reporting. For further information on Nature Portfolio policies, see our Editorial Policies and the Editorial Policy Checklist.

Statistics

For all statistical analyses, confirm that the following items are present in the figure legend, table legend, main text, or Methods section.

n/a	Confirmed	
☐	☒	The exact sample size (*n*) for each experimental group/condition, given as a discrete number and unit of measurement
☐	☒	A statement on whether measurements were taken from distinct samples or whether the same sample was measured repeatedly
☐	☒	The statistical test(s) used AND whether they are one- or two-sided<br>*Only common tests should be described solely by name; describe more complex techniques in the Methods section.*
☒	☐	A description of all covariates tested
☐	☒	A description of any assumptions or corrections, such as tests of normality and adjustment for multiple comparisons
☐	☒	A full description of the statistical parameters including central tendency (e.g. means) or other basic estimates (e.g. regression coefficient) AND variation (e.g. standard deviation) or associated estimates of uncertainty (e.g. confidence intervals)
☐	☒	For null hypothesis testing, the test statistic (e.g. *F*, *t*, *r*) with confidence intervals, effect sizes, degrees of freedom and *P* value noted<br>*Give P values as exact values whenever suitable.*
☒	☐	For Bayesian analysis, information on the choice of priors and Markov chain Monte Carlo settings
☒	☐	For hierarchical and complex designs, identification of the appropriate level for tests and full reporting of outcomes
☒	☐	Estimates of effect sizes (e.g. Cohen's *d*, Pearson's *r*), indicating how they were calculated

Our web collection on statistics for biologists contains articles on many of the points above.

Software and code

Policy information about availability of computer code

Data collection	Data collection is done through a camera mounted on the digital holographic microscope for the holographic measurements. For the atomic force microscopy measurements, we collected the raw data from the AFM device (Nanosurf Flex Axiom) and calculated elasticity coefficients. All image acquisition and processing code related to stiffness calculation was written using the MATLAB® software (version 2018b). Only basic MATLAB functionality was used and most of the code was custom. Fluorescence intensity calculation was done using the ImageJ software (version 1.53). Finite element simulations were done using the COMSOL Multiphysics® software (version 5.3)
Data analysis	Data analysis code was written in MATLAB using only basic MATLAB functionality.

For manuscripts utilizing custom algorithms or software that are central to the research but not yet described in published literature, software must be made available to editors and reviewers. We strongly encourage code deposition in a community repository (e.g. GitHub). See the Nature Portfolio guidelines for submitting code & software for further information.

Data

Policy information about availability of data

All manuscripts must include a data availability statement. This statement should provide the following information, where applicable:

- Accession codes, unique identifiers, or web links for publicly available datasets
- A description of any restrictions on data availability
- For clinical datasets or third party data, please ensure that the statement adheres to our policy

The depth map data used in this study are available in the Zenodo database under https://doi.org/10.5281/zenodo.7197107. Raw holographic recordings were not made public due to their large size (>1TB) but are available upon reasonable request from the corresponding author.

Human research participants

Policy information about studies involving human research participants and Sex and Gender in Research.

Reporting on sex and gender	n/a
Population characteristics	n/a
Recruitment	n/a
Ethics oversight	n/a

Note that full information on the approval of the study protocol must also be provided in the manuscript.

Field-specific reporting

Please select the one below that is the best fit for your research. If you are not sure, read the appropriate sections before making your selection.

☒ Life sciences ☐ Behavioural & social sciences ☐ Ecological, evolutionary & environmental sciences

For a reference copy of the document with all sections, see nature.com/documents/nr-reporting-summary-flat.pdf

Life sciences study design

All studies must disclose on these points even when the disclosure is negative.

Sample size	Five different sample classes were used: polystrene microbeads, poly acrylamide microbeads, agarose microbeads, HCT116 cells and CTC-mimicked HCT116 cells. For all microbeads, the sample size was 50. HCT116 cells were measured using two different methods. For holographically measured HCT116, sample size was 35. For AFM measured HCT116, sample size was 30. For holographically measured CTC-mimicking HCT116 cells, sample size was 25. Sample sizes were determined based on the ability to sufficiently describe their statistical properties. For each independent experiment, at least 5 samples were collected.
Data exclusions	No data were excluded from the study.
Replication	To obtain a measure of the error margin of the proposed method, we first performed some experiments with reference microbeads (Duke Scientific, 2000 Series) with a known stiffness distribution. These experiments were repeated three times. Furthermore, to ensure reproducibility, all holographic experiments were repeated five times. All attempts were successful in the sense that reasonable results (comparable to results reported in the literature) were obtained from every measurement. Furthermore, in order to ensure repeatability we conducted a separate experiment where we measured the same microbeads 10 times consequtively and compared the results. These experiments were done on PS, PAA and agarose microbeads and within DI water, DMEM-F12 and glycerol mediums. As such, there were 9 experiments and 90 measurements in total. These results are presented in the supplementary information file.
Randomization	Experiments were conducted on hollow fluidic chambers. In order to increase randomness of the samples, all samples were selected from a wide range of positions inside the fluidic chamber. As such any bias based on the position of the cell inside the fluidic chip was avoided. Further randomization was deemed uncessary due to the uniform structure of the immortalized cell lines which were used during the experiments.
Blinding	AFM studies were done by a personnel with no knowledge about the sample at hand. As such, he was unaware of the characteristics of the investigated sample. For holographic measurement, no blinding was required since all the measurements were done quantitatively using the same algorithm.

Reporting for specific materials, systems and methods

We require information from authors about some types of materials, experimental systems and methods used in many studies. Here, indicate whether each material, system or method listed is relevant to your study. If you are not sure if a list item applies to your research, read the appropriate section before selecting a response.

Materials & experimental systems

n/a	Involved in the study
☒	☐ Antibodies
☐	☒ Eukaryotic cell lines
☒	☐ Palaeontology and archaeology
☒	☐ Animals and other organisms
☒	☐ Clinical data
☒	☐ Dual use research of concern

Methods

n/a	Involved in the study
☒	☐ ChIP-seq
☒	☐ Flow cytometry
☒	☐ MRI-based neuroimaging

Eukaryotic cell lines

Policy information about cell lines and Sex and Gender in Research

Cell line source(s)	HCT116 cell lines were sourced from American Type Culture Collection (ATCC).
Authentication	The cells used were not authenticated.
Mycoplasma contamination	The cells used were not tested for mycoplasma contamination.
Commonly misidentified lines (See ICLAC register)	No commonly misidentified cell lines were used in this study.
